# Cachexia and Sarcopenia in Patients with Coexisting Chronic Obstructive Pulmonary Disease and Pulmonary Tuberculosis

**DOI:** 10.3390/ijms27208993

**Published:** 2026-10-10

**Authors:** Dmitry Oskin, Stanislav Kotlyarov

**Affiliations:** 1 Department of Infectious Diseases and Phthisiology, Ryazan State Medical University, 390026 Ryazan, Russia; 2 Department of Nursing, Ryazan State Medical University, 390026 Ryazan, Russia; skmr1@yandex.ru

**Keywords:** tuberculosis, chronic obstructive pulmonary disease, post-tuberculosis lung disease, malnutrition, cachexia, sarcopenia, immunometabolism, ceramides, nutritional support, pulmonary rehabilitation

## Abstract

Malnutrition, loss of muscle mass, and reduced muscle strength are common but underrecognized conditions in tuberculosis (TB) and chronic obstructive pulmonary disease (COPD). These abnormalities may persist after completion of TB treatment, and a normal or elevated body mass index does not exclude muscle depletion. Unlike the extensively studied conditions of hyperglycemia and obesity, the catabolic end of the spectrum of metabolic disturbances in coexisting TB and COPD remains insufficiently investigated. This narrative review focuses on cachexia and sarcopenia in patients with coexisting TB and COPD (TB–COPD). It examines the roles of systemic inflammation, immunometabolic remodeling, mitochondrial dysfunction, disturbances in protein metabolism and endocrine regulation, and ceramide signaling. Because direct evidence in TB–COPD is scarce, findings from TB or post-tuberculosis lung disease without confirmed COPD and from COPD alone are used as indirect evidence, and the level of evidence is indicated for each mechanism. The diagnostic approach is organized around current clinical guidelines and includes assessment of nutritional status, diagnosis of malnutrition using the Global Leadership Initiative on Malnutrition (GLIM) criteria, identification of sarcopenia using European Working Group on Sarcopenia in Older People 2 (EWGSOP2) or Asian Working Group for Sarcopenia (AWGS) criteria, pulmonary function testing to distinguish post-tuberculosis airflow obstruction from COPD, and evaluation for reversible causes of muscle weakness. The main treatment approaches are correction of malnutrition, pulmonary rehabilitation, and treatment of the underlying respiratory disease. The effectiveness of interventions is assessed through changes in muscle strength, physical performance, body weight, and body composition. The evidence base for patients with coexisting TB and COPD remains limited; most studies have investigated each disease separately. The efficacy of pharmacological agents targeting the molecular mechanisms of muscle wasting has not been established in this population. Priorities for future research include prospective assessment of muscle function in TB–COPD cohorts and the development of personalized strategies for nutritional and rehabilitation support.

## 1. Introduction

Tuberculosis (TB) and chronic obstructive pulmonary disease (COPD) contribute substantially to global morbidity and mortality. According to World Health Organization (WHO) estimates, approximately 10.7 million people developed TB in 2024, and TB-related deaths among people with and without human immunodeficiency virus (HIV) infection totaled 1.23 million [1]. In 2023, COPD caused approximately 3.4 million deaths, accounting for about 6% of all deaths worldwide [2]. COPD is clinically, etiologically, and biologically heterogeneous and encompasses more than persistent airflow limitation [3].

The relationship between TB and COPD is bidirectional and extends beyond shared risk factors. In a Swedish population-based study, hospitalized patients with COPD had an approximately threefold higher risk of active TB (hazard ratio (HR) 3.0; 95% confidence interval (CI) 2.4–4.0), although smoking data were unavailable in the registries [4]. In a Korean matched cohort, previous TB was associated with subsequent development of COPD (adjusted hazard ratio (aHR), 1.63; 95% CI 1.54–1.73) and COPD-related hospitalization (aHR 2.03; 95% CI 1.81–2.27) [5].

Assessment of coexisting TB and COPD requires consideration of infection activity and the sequence in which the diseases were identified. Persistent airflow obstruction may precede TB, be identified concurrently with it, or be detected after treatment. Post-tuberculosis lung disease (PTLD) also includes restrictive and mixed ventilatory defects, bronchiectasis, fibrosis, and residual cavities; therefore, a history of TB and the presence of airflow obstruction should not automatically be equated with COPD [3,6]. The Global Initiative for Chronic Obstructive Lung Disease (GOLD) taxonomy recognizes tuberculosis-associated COPD within the infection-related etiotype (COPD-I) [7], but in an individual patient the contribution of TB to airflow obstruction can usually be estimated only presumptively (Section 6.3).

Muscle wasting is common in both diseases, but its reported frequency depends on the definition and the setting. In COPD, a meta-analysis of 10 studies (2565 patients) estimated the prevalence of sarcopenia at 21.6% (95% CI 14.6–30.9%), ranging from 8% in population-based to 21% in clinic-based samples and 63% among nursing-home residents [8]; in Asian studies that used Asian Working Group for Sarcopenia (AWGS) criteria, the pooled prevalence was 31% (95% CI 22–41%) [9]. Cachexia was present in 4.6% of 1755 patients with stable COPD [10] and in 4.7% or 10.4% of 1483 patients, depending on whether the consensus or a weight-loss-based definition was applied [11]. In pulmonary TB, undernutrition at diagnosis is frequent in high-burden settings: in a cohort of 1695 adults in rural India, 80% of women and 67% of men had a body mass index below 17.0 kg/m^2^ [12]. Data on sarcopenia in TB are scarcer. Among Korean TB survivors, the prevalence of a sarcopenic phenotype defined mainly by low muscle mass ranged from 10.7% to 24.3%, depending on how muscle mass was normalized [13], and in Malaysian chest clinics, sarcopenia defined using the criteria published by AWGS in 2019 was present in approximately one-third of patients with TB and of those with COPD, compared with 18.9% of patients with asthma [14]. We found no study that reported the prevalence of sarcopenia or cachexia in patients with coexisting TB and COPD (TB–COPD); cohorts that included patients with COPD after TB have reported at most body mass index, recorded obesity, or a composite inflammatory–nutritional index [5,15,16].

Low body weight has clinical significance in its own right. In a recently published meta-analysis of cohort studies, underweight was associated with an increased risk of TB (relative risk (RR), 5.0; 95% CI 4.2–5.9); anthropometric measurements preceded disease onset [17]. In COPD, unintentional weight loss is associated with a poor prognosis regardless of baseline body mass index (BMI) [10]. Weight gain during anti-tuberculosis treatment does not always indicate recovery of muscle tissue: longitudinal studies have shown different changes in fat and fat-free compartments [18,19]. Muscle depletion in COPD can also occur at a normal or elevated BMI [20,21]. Nutritional status, body composition, muscle strength, and exercise tolerance therefore require separate assessment.

Low body weight also appears to modify the link between the two diseases. In a Korean matched cohort, the association between previous TB and incident COPD became stronger as BMI decreased (*p* for interaction = 0.018; Section 3.1) [5]. In a population-based cohort from Eastern China, the association between TB and subsequent COPD was likewise stronger in participants with a lower BMI (*p* for interaction < 0.001) [22], and BMI was among the independent predictors of obstructive disease one year after the diagnosis of pulmonary TB in a prospective Chinese cohort [23]. Similarly, in a Korean COPD cohort, tuberculosis-destroyed lung was more frequent among patients with a low BMI [24]. These studies measured BMI rather than muscle mass or weight trajectories, so they do not show whether weight loss precedes TB–COPD, results from it, or reflects shared risk factors.

Malnutrition, cachexia, and sarcopenia are interrelated but have different diagnostic criteria. Cachexia is a disease-related multifactorial syndrome characterized by loss of muscle mass, with or without loss of fat mass [25]. According to the revised European Working Group on Sarcopenia in Older People (EWGSOP2) consensus, low muscle strength indicates probable sarcopenia, while reduced muscle quantity or quality confirms the diagnosis [26]. Diagnosis of malnutrition using the Global Leadership Initiative on Malnutrition (GLIM) criteria requires a combination of at least one phenotypic and one etiologic criterion [27,28].

Operational definitions of sarcopenia differ between working groups (Table 1). EWGSOP2 cutoffs are derived from European reference populations [26]. AWGS 2019 adopted Asian-specific cutoffs, for example, handgrip strength <28 kg in men and <18 kg in women, and introduced “possible sarcopenia” for case-finding in primary care [29]. The 2025 AWGS consensus update [30] extends diagnostic thresholds to adults aged 50–64 years, defines sarcopenia as the combination of low muscle mass and low muscle strength, treats impaired physical performance as an outcome rather than a diagnostic component, and adds a BMI-adjusted muscle mass index. The Sarcopenia Definition and Outcomes Consortium (SDOC) defined sarcopenia by low grip strength and slow gait speed, without lean mass measured by dual-energy X-ray absorptiometry (DXA) [31], and the Global Leadership Initiative in Sarcopenia (GLIS) proposed a global conceptual definition based on muscle mass, muscle strength, and muscle-specific strength [32]. These differences matter in TB–COPD, because patients are often younger than the older populations in which most cutoffs were derived [15,33,34], and because the reported prevalence of sarcopenia can differ by more than twofold depending on the criteria used [13].

Sarcopenic obesity, the coexistence of excess adiposity with low skeletal muscle mass and function, has its own diagnostic criteria proposed by the European Society for Clinical Nutrition and Metabolism (ESPEN) and the European Association for the Study of Obesity (EASO) [35]. In a cross-sectional study of 198 patients with COPD, its prevalence was 9.6%, and it was associated with more severe dyspnea and worse health-related quality of life [36]. Because obesity and diabetes were recorded less often in patients with COPD and TB or its sequelae than in other patients with COPD [15], the catabolic phenotype may predominate in TB–COPD; nevertheless, a normal or high BMI does not exclude sarcopenic obesity, which can be identified only by assessing body composition and muscle function [35].

The aim of this review is to synthesize the evidence on cachexia and sarcopenia in patients with TB–COPD. Previous reviews have addressed muscle wasting in TB [37] or in COPD [8,38] separately. Because studies that measured muscle mass or strength in patients with both diseases are scarce, we (1) summarize the direct evidence from TB–COPD cohorts, (2) use findings from TB or post-tuberculosis lung disease without confirmed COPD and from COPD alone as indirect evidence and state, for each mechanism and intervention, whether it has been examined in TB–COPD, and (3) propose a practical diagnostic approach that distinguishes post-tuberculosis airflow obstruction from COPD and incorporates the assessment of nutritional status and muscle function.

This work is a narrative review of clinical and experimental studies. The literature search was conducted in English, primarily in PubMed. The literature update was completed on 5 September 2026. Publications were not selected systematically, and results were not quantitatively pooled. Assessment of clinical applicability took into account study design, participant characteristics, and the outcomes investigated. Throughout the review, we indicate whether the evidence was obtained in patients with TB–COPD, in patients with TB or COPD alone, or in experimental models (Section 5). Within TB–COPD, we distinguish active TB with COPD from COPD after previous TB; studies of TB survivors and of post-tuberculosis lung disease without confirmed COPD are treated as indirect evidence.

## 2. Limitations of Conventional Metabolic Assessment in Tuberculosis and Chronic Obstructive Pulmonary Disease

### 2.1. Disturbances in Glucose Metabolism

Metabolic disturbances in TB and COPD are usually considered in two contexts: TB coexisting with diabetes mellitus, and COPD coexisting with obesity and metabolic syndrome. Both approaches are clinically relevant but primarily address hyperglycemia, insulin resistance, and excess adipose tissue. However, loss of body weight and fat-free mass, together with reduced skeletal muscle strength and function, represents another pattern of metabolic disturbances that requires separate assessment.

Coexisting TB and diabetes mellitus have been studied much more extensively than muscle wasting in TB–COPD. In a meta-analysis of 200 studies, the prevalence of diabetes among patients with active TB was 15.3%, but it varied considerably across populations [39]. Cellular and experimental studies show that hyperglycemia and carbonyl stress impair macrophage function and the hypoxia-inducible factor 1 (HIF-1)-dependent antimycobacterial response [40,41]. These studies characterize impaired host defense against infection in hyperglycemia; they did not specifically assess the effects of negative energy balance or muscle wasting.

Hyperglycemia at the diagnosis of active TB does not always indicate preexisting diabetes. In a Tanzanian study, the association between TB and hyperglycemia disappeared after treatment, while a cohort from Dhaka showed changes in glycated hemoglobin (HbA1c) in both directions during treatment [42,43]. These findings support reassessment of glucose metabolism after clinical stabilization. Interpretation of HbA1c must take into account anemia, hemolysis, and other alterations in erythrocyte lifespan [44].

### 2.2. Fat and Fat-Free Mass in Chronic Obstructive Pulmonary Disease

Metabolic syndrome in COPD is associated with abdominal obesity, dysglycemia, and cardiovascular multimorbidity. In the study by Watz et al., it was accompanied by greater systemic inflammation and lower physical activity; however, the cross-sectional design precludes determining the direction of causality [45].

The lower mortality observed in several COPD cohorts among patients with overweight or obesity compared with those with a normal BMI is termed the ‘obesity paradox’ [46,47]. In a secondary analysis of the Study to Understand Mortality and Morbidity in COPD (SUMMIT) involving 16,485 patients with moderate COPD and increased cardiovascular risk, overweight and class I obesity were associated with lower all-cause mortality: HR 0.62 (95% CI 0.52–0.73) and 0.75 (95% CI 0.62–0.90), respectively. No such association was observed in class III obesity (BMI ≥ 40 kg/m^2^); the estimated HR was 1.36 (95% CI 1.00–1.86) relative to normal BMI, defined in the study as 20–<25 kg/m^2^ [47].

This association does not establish a protective effect of adipose tissue. BMI does not distinguish fat mass from fat-free mass, whereas a low fat-free mass index has independent prognostic significance in COPD [20]. Smoking, disease severity, and weight loss before study enrollment may influence the findings: a lower BMI may be a consequence of progressive disease [47]. The ‘obesity paradox’ therefore does not justify omitting assessments of muscle strength and body composition in patients with a high BMI [20,21].

In the Copenhagen City Heart Study, which included 1898 patients with COPD, 26.1% of participants with a normal BMI had a fat-free mass index below the 10th percentile of the general population. Across the cohort, a low fat-free mass index was associated with all-cause mortality (HR 1.5; 95% CI 1.2–1.8) and COPD-related mortality (HR 2.4; 95% CI 1.4–4.0), independently of several clinical factors [20]. In the COPD and Systemic Consequences–Comorbidities Network (COSYCONET) cohort, a low fat-free mass index was identified across BMI categories, including class I obesity; BMI-adjusted reference values were used, so these frequencies cannot be directly compared with those from studies applying a fixed cutoff [21]. Fat-free mass includes tissues other than muscle, which further limits its interpretation.

### 2.3. Mixed Metabolic Disturbances

Hyperglycemia and muscle wasting are not mutually exclusive. In a Korean cohort, the combination of diabetes and underweight was associated with a higher risk of TB than either characteristic alone [48]. Sarcopenic obesity, in turn, involves the coexistence of excess adipose tissue, reduced muscle mass, and impaired muscle function [35]. Thus, neither normal glycemia nor a high BMI excludes malnutrition or muscle depletion [26,27,35].

The pattern of recorded comorbidities may differ when COPD coexists with TB or its sequelae. In a retrospective population-based cohort, obesity, diabetes mellitus, and arterial hypertension were less common among patients with TB or post-tuberculosis changes than among the remaining patients with COPD [15]. These differences characterize the frequency of recorded diagnoses but do not indicate whether muscle tissue is preserved or cachexia is present. Without measurements of body composition and muscle strength, a lower frequency of diabetes and obesity cannot be interpreted as a favorable metabolic profile.

For metabolic stratification of patients with TB–COPD, it is useful to distinguish two relatively independent dimensions: excess adipose tissue and insulin resistance on the one hand, and the extent of catabolism with muscle wasting on the other. Their coexistence gives rise to mixed conditions, including diabetes mellitus in patients with underweight and sarcopenic obesity. This approach helps avoid incorrectly classifying all patients without diabetes as metabolically healthy.

**Figure 1:**
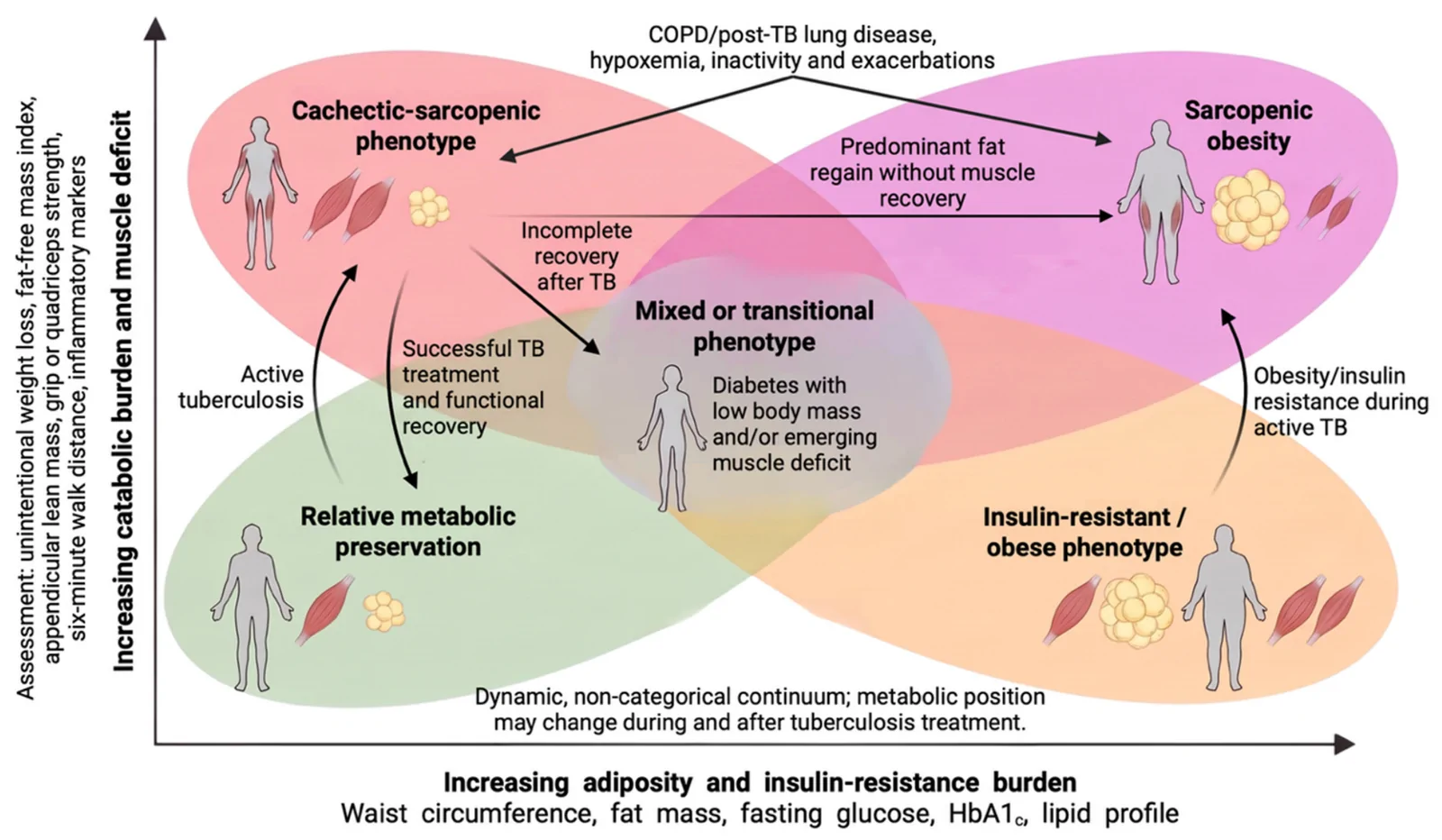
Metabolic space in coexisting tuberculosis and COPD. The horizontal axis represents increasing adiposity and insulin resistance, whereas the vertical axis represents increasing catabolic burden and muscle deficit. Colored regions illustrate overlapping metabolic states, and arrows indicate possible changes during active tuberculosis and after treatment completion. This space is conceptualized as a dynamic continuum. The schematic is descriptive and does not define fixed diagnostic boundaries or constitute a diagnostic classification. Created in BioRender. Oskin, D. (2026) https://BioRender.com/1kcsyix. Abbreviations: COPD, chronic obstructive pulmonary disease; HbA1_c_, glycated hemoglobin; TB, tuberculosis.

A patient’s position along both axes of this metabolic space may change over time. During active TB, a patient with obesity and insulin resistance may develop muscle depletion while retaining excess adipose tissue. After treatment, partial recovery may occur, but fat regain may outpace muscle recovery, and muscle depletion and weakness may persist. Accordingly, a single assessment based on BMI or diabetes status does not capture the patient’s metabolic trajectory. This framework, illustrated in Figure 1, describes a continuum of changing metabolic states and does not constitute a diagnostic classification.

## 3. Clinical Manifestations and Prognostic Significance of Cachexia and Sarcopenia in Tuberculosis, Chronic Obstructive Pulmonary Disease, and Their Coexistence

A single measure is insufficient for clinical assessment of catabolic and sarcopenic abnormalities in TB–COPD. A low BMI alone does not indicate cachexia, and reduced fat-free mass without assessment of muscle strength does not establish a diagnosis of sarcopenia. Reduced physical performance is also nonspecific: it may be related to ventilatory impairment, hypoxemia, cardiovascular disease, or overall disease severity. These limitations are reflected in the definitions of cachexia, sarcopenia, and malnutrition [25,26,27].

### 3.1. Evidence on Coexisting Tuberculosis and Chronic Obstructive Pulmonary Disease

Studies directly investigating coexisting TB and COPD, mostly in patients with COPD after previous TB, show higher hospitalization rates and, in some cohorts, increased mortality [5,15,33,34]. However, these studies primarily address clinical outcomes: nutritional status was assessed in only a subset of studies, and data on muscle mass and strength are almost entirely absent.

In a retrospective cohort of 16,714 patients with COPD, TB or its sequelae were recorded in 267 individuals (1.60%). Of these, 73.8% had post-tuberculosis changes, 8.2% had active TB, and a further 18.0% had both. Patients in this group were younger, were predominantly male, and had higher mortality than the remaining patients with COPD: 16.5% versus 10.6% (*p* = 0.003). The association with mortality persisted after adjustment for the factors considered (adjusted odds ratio (aOR), 1.61; 95% CI 1.14–2.28). The risk of exacerbations remained elevated after adjustment for age and sex (aOR 1.43; 95% CI 1.05–1.96), but the association lost statistical significance after additional adjustment for post-tuberculosis structural changes. TB or its sequelae were associated with bronchiectasis (odds ratio (OR), 6.07; 95% CI 3.03–12.16), pulmonary fibrosis (OR 5.67; 95% CI 3.40–9.45), and pneumonia (OR 2.01; 95% CI 1.50–2.71) [15].

In a Korean study, 31,033 TB survivors were compared with the same number of participants without TB, matched for age and sex. Screening data were obtained predominantly within the two years before TB diagnosis. Even during this period, individuals who subsequently developed TB had a lower mean BMI (22.1 ± 2.9 versus 24.1 ± 3.1 kg/m^2^; *p* < 0.001), a fourfold higher prevalence of underweight (9.0% versus 2.2%), and lower physical activity (577 ± 614 versus 648 ± 631 metabolic equivalent of task (MET)-minutes per week; *p* < 0.001). Among participants who subsequently developed COPD, the risk of COPD-related hospitalization after TB was higher than in those with COPD without a history of TB (aHR 1.84; 95% CI 1.17–2.92) [5]. The association between previous TB and the development of COPD became stronger as BMI decreased (*p* for interaction = 0.018), suggesting that baseline nutritional status may modify the magnitude of risk. No such interaction was identified for hospitalization (*p* = 0.703). Because measurements preceded TB diagnosis, they characterize anthropometric status and physical activity before the disease, rather than loss of muscle tissue after infection [5].

Population-based data are complemented by several small clinical studies. Among 74 patients with COPD, 24 (32.4%) had a history of pulmonary TB. Despite a comparable degree of airflow obstruction, these patients were younger, had lower cumulative smoking exposure (*p* = 0.027), and were hospitalized more frequently (*p* = 0.01) [33]. In another sample of 598 patients hospitalized with a COPD exacerbation, 93 (15%) had a history of TB. In this group, the mean age at COPD diagnosis was four years lower and the mean age at death was five years lower; the number of hospitalizations was also higher: 2.46 ± 0.26 versus 1.56 ± 0.88 per year (*p* = 0.001). However, the difference in three-year survival did not reach statistical significance (*p* = 0.08), and previous TB was not an independent predictor of mortality in the Cox model [34]. These studies support the clinical vulnerability of patients with TB–COPD but do not establish whether muscle wasting causes a poor prognosis, mediates it, or merely marks disease severity. Additional evidence on the relationship between residual lung disease, inflammation, and nutritional status comes from a retrospective study of 2210 patients with previous TB. A composite inflammatory–nutritional index was associated with the development of radiologically detected bronchiectasis, and coexisting COPD was independently associated with this outcome. These findings do not directly characterize muscle tissue but indicate that inflammatory, nutritional, and structural lung changes should be assessed together [16].

### 3.2. Catabolic Disturbances in Active Tuberculosis

Weight loss, reduced appetite, and generalized weakness are characteristic systemic manifestations of active TB [12,49]. They cannot be explained solely by inadequate access to food: studies have identified alterations in the hormonal regulation of appetite, energy metabolism, and the anabolic response to feeding [50,51,52]. These factors may jointly limit restoration of the body’s protein stores. Appetite impairment in active TB is associated, in part, with altered hormonal and inflammatory signals [50,52]. A comparison of 41 patients with newly diagnosed pulmonary TB and 82 healthy participants showed higher levels of peptide YY, ghrelin, and resistin and lower leptin concentrations in the patients. During treatment, these measures approached the values observed in the control group. Appetite decreased as peptide YY levels increased, and peptide YY was the strongest independent predictor of appetite at all follow-up assessments. A high baseline concentration also predicted smaller improvements in appetite and smaller gains in fat mass during treatment [50]. However, the small sample size and observational design preclude considering this mechanism universal in TB-associated cachexia.

A study using labeled leucine demonstrated an impaired postprandial anabolic response. Fasting protein turnover was not increased in nine patients with pulmonary TB, in contrast to six undernourished participants without TB. After feeding, the proportion of leucine oxidized was higher in patients with TB than in either comparison group (33.2% versus 24.0% in each of the two comparison groups), and their protein balance was the least positive. The authors termed this an ‘anabolic block’ [51]. Comparison with undernourished individuals without infection indicates that disturbances in protein metabolism in TB are related not only to inadequate food intake but also to an impaired response to feeding.

Body composition studies support the prognostic significance of wasting. In a Ugandan cohort of 747 patients with pulmonary TB, associations with mortality differed between men and women. In men, a low BMI was an adverse prognostic factor (HR 1.7; 95% CI 1.03–2.81), whereas the fat-free mass index had no prognostic significance. In women, by contrast, a low fat-free mass index was associated with mortality (HR 5.0; 95% CI 1.6–15.9), while the association with BMI did not reach statistical significance [53]. Assessment of body composition may therefore provide prognostic information that BMI does not always capture. However, the pronounced sex differences, high prevalence of HIV infection, and use of bioelectrical impedance analysis preclude direct extrapolation of these findings to patients with TB–COPD.

In a retrospective study of 309 patients with active TB, the area under the receiver operating characteristic (ROC) curve was 0.761 for the skeletal muscle index at the level of the twelfth thoracic vertebra (T12) and 0.700 for BMI. However, the difference between the ROC curves did not reach statistical significance. This finding suggests potential prognostic value for computed tomography (CT)-based muscle assessment, requires external validation, and does not replace measurement of muscle function [54].

### 3.3. Recovery of Body Composition After Tuberculosis Treatment

Weight gain during anti-tuberculosis therapy is traditionally considered a favorable sign [55]. Assessment of recovery requires consideration of changes in both fat and fat-free compartments [18,19,49]. An increase in fat mass despite persistent muscle depletion may give the impression of recovery even though muscle weakness and functional limitations remain.

In a longitudinal study, body composition was assessed using dual-energy X-ray absorptiometry and a four-component model. Over six months of treatment, body weight in 30 patients increased by approximately 10% and fat mass by 44%. Protein mass, total body water, and bone mineral mass, however, did not change substantially [18]. Thus, microbiological cure and recovery of body weight are not necessarily accompanied by restoration of the body’s protein stores.

In a prospective Ugandan cohort, body composition in 717 patients was assessed before treatment and subsequently at 3, 12, and 24 months. The pattern of recovery depended on sex and the baseline degree of wasting. In men with wasting, fat-free mass increased faster than fat mass during the first months of treatment, whereas in women with wasting, weight gain occurred predominantly through accumulation of fat tissue. The most pronounced changes occurred in the first three months, after which recovery slowed considerably, and some patients remained underweight [19]. Comparisons across studies also require consideration of HIV status and the method used to measure body composition.

### 3.4. Muscle Mass and Physical Function After Tuberculosis

Evidence linking previous TB to persistently reduced muscle mass comes from population-based studies using dual-energy X-ray absorptiometry. Among 3228 Korean men aged 50 years or older, the combination of a history of TB and residual radiographic changes was associated with more than a threefold increase in the odds of sarcopenia (OR 3.44; 95% CI 1.79–6.68). Radiographic post-tuberculosis changes without a documented history of TB were also associated with higher odds of sarcopenia (OR 2.05; 95% CI 1.05–4.00) [56]. These findings suggest that the risk of muscle depletion is greater with more pronounced PTLD, although this study design does not establish causality.

Another analysis of the Korea National Health and Nutrition Examination Survey (KNHANES) data included 9203 people aged 40 years or older. Among TB survivors, the prevalence of the sarcopenic phenotype was 11.2%, 10.7%, or 24.3%, depending on whether appendicular muscle mass was normalized to BMI, total body weight, or height squared. With each calculation method, prevalence was higher than in people without TB. Adequate protein and energy intake was associated with a lower risk of muscle depletion [13]. The more than twofold difference between the estimates illustrates how strongly the reported prevalence of sarcopenia depends on the criteria selected. Moreover, in both Korean studies, diagnosis was based predominantly on muscle quantity, without mandatory assessment of strength. Under EWGSOP2, these findings more accurately describe reduced muscle mass than confirmed sarcopenia [26].

Studies of physical performance complement body composition data. In 28 patients with active TB during the intensive phase of treatment, the six-minute walk distance and peak oxygen uptake were substantially lower than in 19 healthy individuals of comparable age: 571.7 ± 121.0 versus 841.6 ± 53.0 m and 18.1 ± 2.8 versus 24.3 ± 1.2 mL∙kg^−1^∙min^−1^, respectively. Both measures improved markedly after two months of therapy [57]. In 60 patients aged 50–65 years with post-tuberculosis lung changes, the six-minute walk distance and estimated maximal oxygen uptake were also lower than in 60 healthy participants of the same age [58]. However, the six-minute walk test cannot isolate the contribution of skeletal muscles: its result depends on the combined performance of the respiratory, cardiovascular, and muscular systems.

Reduced peripheral muscle strength has also been identified in PTLD. In a study of 47 patients, handgrip strength was reduced in 34% of participants and quadriceps strength in 25.5%. Lower strength in both muscle groups and reduced pulmonary diffusing capacity were independently associated with poorer performance on the Glittre test, which assesses functional performance in activities of daily living. Together, these measures explained 39% of the variability in test completion time. The cross-sectional design precludes determining the direction of causality, but the findings show that the functional consequences of previous TB extend beyond abnormalities detected by spirometry [59].

### 3.5. Muscle Wasting in Chronic Obstructive Pulmonary Disease

The clinical significance of muscle wasting in COPD, irrespective of TB history, has been studied much more extensively. Low fat-free mass is identified across BMI categories, including in patients with normal or increased body weight [20,21,60]. Its clinical significance is assessed through its relationships with muscle strength, physical performance, and prognosis [38,61].

Among 622 outpatients with stable COPD, sarcopenia defined by the original European Working Group on Sarcopenia in Older People (EWGSOP) criteria, which incorporate muscle mass and function, was identified in 14.5% (95% CI 11.8–17.4). Patients with sarcopenia had poorer exercise tolerance, were less active, and had worse health status. Nevertheless, they responded to pulmonary rehabilitation as well as patients without sarcopenia; after the program, 12 of 43 participants no longer met the diagnostic criteria [62]. These findings demonstrate the potential for functional improvement but were obtained outside the context of active TB.

In COPD, measures of muscle mass and strength complement BMI-based prognostic assessment. In a study of 142 patients, the combination of forced expiratory volume in one second (FEV_1_) <50% predicted and mid-thigh muscle area <70 cm^2^ was associated with an increased risk of death relative to the group with both measures above these thresholds (HR 13.16; 95% CI 1.74–99.20); the wide interval reflects limited precision of the estimate [63]. In another cohort of 162 patients, greater quadriceps strength normalized to BMI was associated with a lower risk of the composite outcome of death or lung transplantation [64]. These observations should not be interpreted as estimates of the effect of treatment-induced gains in strength.

In a prospective cohort of 1755 patients with stable COPD, cachexia and precachexia were present in 4.6% and 1.6% of participants, respectively. After multivariable adjustment, unintentional weight loss predicted mortality (aHR 2.160; 95% CI 1.515–3.079), whereas a low fat-free mass index without weight loss did not [10]. A single weight measurement therefore cannot substitute for assessment of weight change over time.

Dyspnea, low physical activity, and muscle weakness mutually reinforce functional limitations in COPD [38,61,65]. Exacerbations and hospitalizations create additional periods of inflammation, poorer nutrition, and immobilization [66]. In patients with previous TB, these factors may hinder recovery; however, the contribution of each factor and the independent prognostic significance of muscle depletion specifically in TB–COPD require direct investigation [6,15,67].

### 3.6. Muscle Wasting in Tuberculosis, Chronic Obstructive Pulmonary Disease, and Their Coexistence: Similarities and Differences

No study has directly compared muscle mass, muscle strength, or their prognostic value among patients with TB–COPD, TB alone, and COPD alone. The available data describe different trajectories of muscle loss in TB and in COPD (Table 2). In active TB, wasting is typically present at diagnosis, is accompanied by anorexia, systemic inflammation, and an anabolic block, and partly reverses during treatment, although fat mass recovers faster than protein mass [18,19,50,51]. In COPD, muscle loss usually develops gradually and is accentuated by exacerbations and hospitalizations, after which recovery is incomplete [38,66]. The trajectories observed in TB and COPD may overlap when the diseases coexist; this has not been directly studied.

Direct data on TB–COPD come mainly from patients with COPD after previous TB. These studies reported more frequent hospitalizations and, in some cases, exacerbations than in other patients with COPD [5,15,33,34], higher circulating C-reactive protein (CRP) and interleukin 6 (IL-6) levels than in smoking-related COPD [68], and less frequently recorded obesity and diabetes [15]; in a general COPD cohort, tuberculosis-destroyed lung was more common among patients with a low BMI [24]. Data on active TB with COPD are scarce: in one COPD cohort, a code for active TB was recorded at some time during the observation period in about a quarter of the patients with TB or its sequelae, but these patients were analyzed together with those who had post-tuberculosis changes only [15].

Indirect data come from TB survivors, from patients with post-tuberculosis lung disease without confirmed COPD, and from comparisons of separate TB and COPD groups. Among Korean men aged 50 years or older, the odds of sarcopenia, defined by muscle mass measured by DXA, were increased in those with both a history of TB and residual radiographic changes (OR 3.44; 95% CI 1.79–6.68), and radiographic changes without a reported history of TB were also associated with it (OR 2.05; 95% CI 1.05–4.00) [56]; residual lung damage may therefore be relevant beyond the history of infection itself. In a cross-sectional study of chest-clinic patients treated for TB or for COPD, in which patients with both diseases were not analyzed separately, sarcopenia defined by AWGS 2019 criteria was similarly frequent in both groups (approximately one-third), and lower BMI was associated with sarcopenia in both groups [14]. Whether sarcopenia or cachexia carries greater prognostic weight in TB–COPD than in either disease alone has not been studied; the prognostic estimates available for TB [53,54] and for COPD [10,20,63,64] cannot be combined or transferred to this population. Prospective cohorts are needed that include patients with active TB and COPD, patients with COPD after TB, and TB-only and COPD-only comparison groups, with standardized measurement of muscle mass and strength (Section 7.6).

**Table 2:** Muscle wasting in TB, COPD, and TB–COPD: summary of the available evidence. In the TB–COPD column, direct data come from patients with COPD after previous TB or with active TB and COPD; indirect data come from TB survivors or patients with post-tuberculosis lung disease without confirmed COPD.

Feature	TB (Active and After Treatment)	COPD	TB–COPD
Main drivers	Anorexia and altered appetite hormones, systemic inflammation, anabolic block [50,51,52]	Systemic and muscle inflammation, physical inactivity, hypoxemia, exacerbations, glucocorticoids [38,66,69]	Not directly studied; drivers of both diseases may combine. Direct (COPD after TB): higher CRP and IL-6 than in smoking-related COPD [68]. Indirect (TB survivors): residual structural damage [56]
Time course	Present at diagnosis; partial recovery during treatment, with fat regain exceeding protein regain [18,19]	Gradual, with stepwise losses during exacerbations [38,66]	Not studied; the trajectories of TB and COPD may overlap
Frequency	Undernutrition (BMI < 17.0 kg/m^2^) in 67–80% at diagnosis in a high-burden setting [12]; sarcopenia in about one-third of chest-clinic patients [14]; low muscle mass in 10.7–24.3% of survivors [13]	Sarcopenia in 21.6% (meta-analysis) [8] and 31% in Asian AWGS-based studies [9]; cachexia in 4.6–10.4%, depending on the definition [10,11]	No data on sarcopenia or cachexia
Prognostic data	Low fat-free mass index and mortality in women [53]; CT-based muscle index and prognosis [54]	Weight loss, fat-free mass index, thigh muscle area, and quadriceps strength predict mortality [10,20,63,64]	Direct, mainly COPD after TB: hospitalizations, exacerbations, and mortality reported without muscle measures [5,15,33,34]
Response to intervention	Nutritional support improves weight gain and probably handgrip strength [70,71]	Pulmonary rehabilitation improves function also in sarcopenia [62]; nutritional supplements add little to muscle mass [72,73]	No trials in TB–COPD. Indirect: rehabilitation improved exercise capacity in post-tuberculosis lung disease without confirmed COPD [74]

Abbreviations: AWGS, Asian Working Group for Sarcopenia; BMI, body mass index; COPD, chronic obstructive pulmonary disease; CRP, C-reactive protein; CT, computed tomography; IL-6, interleukin 6; TB, tuberculosis; TB–COPD, coexisting tuberculosis and chronic obstructive pulmonary disease.

Thus, muscle wasting is associated with adverse outcomes in TB and in COPD, but whether muscle depletion is an independent cause of these outcomes or a marker of disease severity remains unclear, and its prognostic effect in patients with both diseases has not been examined. Section 4 considers the mechanisms that may explain these clinical observations, beginning with the limited evidence obtained in TB–COPD.

## 4. Pathophysiological Mechanisms of Muscle Wasting

Muscle wasting may be associated with systemic inflammation, immunometabolic and bioenergetic reprogramming, mitochondrial dysfunction, and impaired proteostasis and endocrine regulation. Hypoxemia, insufficient physical activity, smoking, and drug exposure may exacerbate these processes [38,51,75,76,77].

Most mechanistic studies have examined TB or COPD separately. The findings below are presented with reference to the cells, tissues, and clinical conditions studied; their combined effects in patients with coexisting diseases remain under investigation [51,75,76,77]. Section 4.1 summarizes the observations obtained in patients with TB–COPD, mainly COPD after previous TB, and, separately, the indirect evidence from TB survivors and post-tuberculosis lung disease without confirmed COPD; the subsequent subsections indicate whether each mechanism is supported by human data in TB or COPD or only by experimental models, and Section 5 gives the level of evidence.

### 4.1. Evidence Specific to Coexisting Tuberculosis and Chronic Obstructive Pulmonary Disease

No study has examined skeletal muscle biology in patients with TB–COPD, and no study has analyzed active TB with COPD separately. Several observations, obtained mainly in patients with COPD after previous TB, nevertheless point to processes that are known to promote muscle wasting. In the studies cited here, TB-associated COPD was defined as airflow obstruction after TB with extensive parenchymal destruction and without COPD before TB [68,78]; this group therefore overlaps with post-tuberculosis airflow obstruction as described in Section 6.3. In TB-associated COPD, systemic inflammation appeared more pronounced than in smoking-related COPD: CRP, erythrocyte sedimentation rate, and IL-6 levels were higher, and IL-6 predicted exacerbations only in TB-associated COPD [68]. In a cross-sectional study of 737 patients with previous or current TB, the systemic inflammatory response index, calculated from blood cell counts, distinguished the 83 patients with TB-associated obstructive pulmonary disease from those without airflow obstruction with moderate accuracy (area under the receiver operating characteristic curve, 0.702 before propensity score matching) [79]; because TB activity was not reported separately, this study may have included patients with active TB. Signaling through IL-6 and signal transducer and activator of transcription 3 (STAT3) can drive muscle atrophy in experimental models [80], so persistently elevated IL-6 is a plausible, although untested, link between TB–COPD and muscle loss. The serum metabolome also differs between TB-associated and smoking-related COPD, with higher kynurenine and lower serotonin concentrations, consistent with increased tryptophan degradation through indoleamine 2,3-dioxygenase [78]. In older adults, kynurenine concentrations correlated with IL-6, weaker grip strength, slower gait, and frailty [81]; this pathway has not been studied in relation to muscle in TB–COPD. Hospitalizations and, in some studies, exacerbations are also more frequent in COPD after TB (Section 3.6); because quadriceps thickness declines during hospitalization for an exacerbation and recovers only partly [66], this may add a further catabolic stimulus, although muscle was not measured in these cohorts.

Indirect evidence comes from TB survivors and from post-tuberculosis lung disease without confirmed COPD. In a retrospective study of 2210 patients with previous TB, a composite inflammatory–nutritional index was associated with the development of radiologically detected bronchiectasis, and coexisting COPD was independently associated with the same outcome [16]. In adults followed from the diagnosis of pulmonary TB, enhanced interferon-related transcriptional responses at the end of treatment were associated with obstructive post-tuberculosis lung disease six months later [82]. In immune myopathies, interferon-γ (IFN-γ) caused myogenic cell dysfunction and senescence [83]; whether persistent interferon signaling after TB affects skeletal muscle is unknown. Residual structural damage in TB survivors may add a further catabolic stimulus (Section 3.6) [56]. Together, these observations suggest that the catabolic drivers of TB and COPD may converge in TB–COPD, but the evidence linking them to muscle wasting is indirect. The studies of COPD after TB did not report measurements of muscle mass, strength, or muscle tissue; muscle mass was measured only in TB survivors without confirmed COPD [13,56]; and no study has analyzed active TB with COPD separately.

### 4.2. Inflammatory and Stress Signals

The inflammatory response in TB should not be regarded as uniformly harmful. COPD and pulmonary TB share innate and adaptive immune mechanisms that may sustain inflammation and contribute to lung tissue injury when these diseases coexist [84]. IFN-γ-dependent macrophage activation contributes to the control of *Mycobacterium tuberculosis* [85], while the development of TB during tumor necrosis factor (TNF) blockade indicates the importance of this cytokine in host defense against infection [86]. During prolonged inflammation, these same signals may contribute to systemic catabolism [69,80,87,88].

In cultured muscle cells, TNF activated the canonical nuclear factor kappa-light-chain-enhancer of activated B cells (NF-κB) pathway and reduced the expression of inhibitor of nuclear factor kappa B kinase subunit alpha (IKK-α), a regulator of the muscle oxidative phenotype [89]. In a transgenic mouse model, activation of inhibitor of nuclear factor kappa B kinase subunit beta (IKKβ)/NF-κB increased ubiquitin-dependent proteolysis and expression of the E3 ubiquitin ligase muscle RING finger protein 1 (MuRF1), causing marked muscle atrophy [90]. These findings describe a possible mechanism of inflammatory catabolism but do not establish its contribution in patients with coexisting TB and COPD. In a placebo-controlled pilot study of 30 men with active TB, including both HIV-positive and HIV-negative participants, thalidomide administered in 14-day cycles reduced TNF production and was associated with greater weight gain than placebo [88]. This finding is consistent with an association between TNF and TB-associated wasting but does not establish a specific causal role for this cytokine: the sample was small, follow-up was short, and the drug had multiple immunomodulatory effects. In the same study, IFN-γ production increased during thalidomide treatment [88].

Evidence of inflammatory activation within skeletal muscle itself has been reported in COPD. A comparison of quadriceps biopsy specimens from seven patients with a low BMI (mean, 18.5 kg/m^2^) and seven patients who were not underweight (mean BMI, 27.5 kg/m^2^) showed 30% higher NF-κB deoxyribonucleic acid (DNA)-binding activity, reduced content of the inhibitor of nuclear factor kappa-B alpha (IκBα) , and higher inducible nitric oxide synthase (iNOS) expression in the former group [69]. NF-κB activation may simultaneously contribute to proteolysis, nitrosative stress, and loss of the oxidative phenotype of muscle fibers. The very small sample and cross-sectional design preclude determining whether NF-κB activation precedes muscle mass loss or reflects established cachexia.

IL-6 exerts systemic effects through classical signaling and trans-signaling involving glycoprotein 130 (gp130), Janus kinase (JAK), and predominantly STAT3. Sustained JAK/STAT3 activation can induce the hepatic acute-phase response, alter amino acid metabolism, and increase atrogene expression. In experimental models with high IL-6 levels, STAT3 activation was necessary and sufficient for muscle atrophy, while pharmacological or genetic inhibition of JAK/STAT3 reduced muscle tissue loss [80]. These findings were obtained mainly in models of cancer cachexia and sterile inflammation; direct evidence that this pathway contributes to muscle atrophy in coexisting TB and COPD is lacking.

The acute-phase response increases the hepatic demand for amino acids to synthesize CRP, serum amyloid A, fibrinogen, and other proteins. When protein intake is inadequate, skeletal muscle may serve as a source of these amino acids. An attenuated postprandial anabolic response and an increased proportion of leucine undergoing oxidation have been demonstrated in active TB [51]. These findings are consistent with possible amino acid redistribution, although its quantitative contribution to muscle mass loss in TB–COPD has not been determined. A causal role for the IL-6–JAK–STAT3 pathway in atrophy has been established predominantly in experimental models [80].

Circulating cytokines do not provide a universal explanation for muscle wasting. In a three-year study in COPD, tumor necrosis factor alpha (TNF-α) was associated with fat-free mass loss only in patients with established cachexia [87]. This finding limits the use of serum cytokines as standalone predictors but does not exclude local inflammatory effects on muscle.

In addition to classical proinflammatory cytokines, mediators of cellular and mitochondrial stress may participate in the regulation of appetite and energy metabolism. The most extensively studied of these is growth differentiation factor 15 (GDF-15). Its metabolic effects are mediated through the glial cell line-derived neurotrophic factor family receptor alpha-like (GFRAL) and the rearranged during transfection (RET) receptor tyrosine kinase coreceptor, with GFRAL expression largely restricted to hindbrain structures. In experiments in mice, GDF-15 administration not only reduced food intake but also prevented the decrease in energy expenditure that usually accompanies caloric restriction; this effect was associated with increased energy metabolism in skeletal muscle [91]. In patients with acute respiratory failure, lower airway GDF-15 levels correlated with plasma levels; causal evidence of GDF-15 secretion by the pulmonary epithelium was obtained in a mouse model of acute injury induced by *Pseudomonas aeruginosa* exoproducts [92]. In a mouse model of aging, increased *Gdf15* expression in skeletal muscle was accompanied by impaired mitochondrial respiration and higher circulating GDF-15 concentrations [93]. These findings suggest that the pulmonary epithelium and skeletal muscles may contribute to circulating GDF-15 during cellular stress, but they do not establish these tissues as the main sources of this protein in patients with TB or COPD.

Clinical studies of GDF-15 are predominantly observational. Elevated concentrations of this protein were associated with a greater likelihood of sarcopenia and poorer physical performance in population samples [94] and with lower muscle mass, handgrip strength, and quadriceps strength in patients with stable COPD [95]. During COPD exacerbations, higher GDF-15 concentrations were associated with malnutrition and adverse nutritional status indicators [96]. GDF-15 is considered alongside other mediators of mitochondrial stress, particularly fibroblast growth factor 21 (FGF-21) and humanin. In stable COPD, concentrations of these proteins showed differing relationships with exercise tolerance and exacerbation risk [97], while in older adults, GDF-15 and FGF-21 were associated with frailty, sarcopenia, and malnutrition [98]. However, GDF-15 is not a specific marker of muscle wasting: its concentration depends on age, comorbidities, the characteristics of the population studied, and the laboratory assay used. The thresholds proposed in individual studies therefore cannot yet be considered universal, particularly for patients with coexisting TB and COPD.

Elevated GDF-15 concentrations may also be associated with smoking. In an observational study, smoking was associated with higher GDF-15 concentrations and a lower body mass index. Mediation analysis and Mendelian randomization suggested that GDF-15, particularly its ratio to RET, may partly contribute to smoking-related changes in body weight. However, the mediation analysis results differed according to the proteomic platform used [99]. The trial of the GDF-15 antibody ponsegromab in cancer cachexia is discussed in Section 7.6 [100].

### 4.3. Immunometabolic Reprogramming

Macrophage activation requires the reprogramming of energy metabolism. Hypoxia-inducible factor 1 alpha (HIF-1α) supports IFN-γ-dependent anti-TB activity, while HIF-1α-dependent induction of lactate dehydrogenase A (LDHA) directs pyruvate toward lactate production and limits its availability to intracellular mycobacteria [85,101]. Increased glycolysis in this setting therefore represents a protective response rather than metabolic dysfunction in itself.

The response to mycobacteria is not limited to a unidirectional increase in glycolysis. Concurrent decreases in glycolytic activity and oxidative phosphorylation, with a shift toward slower cellular energy metabolism, have been described in human macrophages infected with *M. tuberculosis* [102]. The direction of metabolic changes depends on the cellular model and the conditions of infection.

The axis involving adenosine monophosphate-activated protein kinase (AMPK) and mechanistic target of rapamycin (mTOR) links cellular energy status, autophagy, and cell growth. AMPK is activated when the adenosine monophosphate (AMP)/adenosine triphosphate (ATP) ratio increases, promoting catabolic processes and autophagy while suppressing energy-intensive biosynthetic processes through mechanistic target of rapamycin complex 1 (mTORC1). In myeloid cells, the AMPK–mTOR axis involving transcription factor EB (TFEB) regulates lysosomal function and autophagy, thereby influencing the ability of the cell to control intracellular *M. tuberculosis* [103]. The role of mTOR in this context is complex and depends on the specific conditions. In experimental models of mycobacterial infection, mTORC1 activity supported mitochondrial energy metabolism and the viability of infected macrophages, reducing mitochondrial damage associated with the 6-kDa early secretory antigenic target (ESAT-6) and cell death [104]. In skeletal muscle, by contrast, a sustained decrease in mTORC1 activity limits protein synthesis and contributes to atrophy [76,105].

The cell specificity of these effects is important when interpreting the findings: mTORC1-mediated maintenance of metabolism in an infected macrophage and regulation of muscle protein synthesis are distinct processes. Findings from interventions targeting AMPK–mTOR in immune cells are therefore insufficient to guide the selection of treatment for muscle wasting [76,103,104,105,106].

Tricarboxylic acid cycle intermediates also regulate the immune response. In experiments with *Mycobacterium bovis*, disruption of fumarate reductase (*frd*) operon function reduced succinate secretion and the protective response dependent on HIF-1α and interleukin 1 beta (IL-1β) while increasing pathogenicity [107]. In another model of mycobacterial infection, excess TNF sustained mitochondrial reactive oxygen species (ROS) production through reverse electron transport [108]. The effects of succinate are therefore context-dependent; these findings do not establish its role in muscle catabolism in TB–COPD.

Itaconate is another metabolite involved in the regulation of the immune response. It can directly inhibit several *M. tuberculosis* enzymes, although its production varies substantially across animal species and macrophage types. In particular, human alveolar macrophages produce considerably less itaconate than mouse macrophages. In addition, sustained elevation of aconitate decarboxylase 1 (ACOD1) activity may compromise lysosomal membrane integrity. The metabolic state of the macrophage also affects the tolerance of intracellular mycobacteria to anti-TB drugs. For example, serine availability alters the balance between AMPK and mTOR signaling, the extent of autophagy, and the ability of the macrophage to restrict mycobacterial growth [109,110,111,112,113,114].

Changes in cellular metabolism may persist after the initial stimulus has ceased. Redistribution of tricarboxylic acid cycle enzymes and changes in the abundance of its intermediates were associated with DNA hypermethylation and the development of an immune-tolerant state in monocytes after exposure to lipopolysaccharide or *M. tuberculosis*. These findings suggest that immunometabolic changes may persist long after infection. Their relationship with muscle mass loss and increased catabolism in humans, however, has not yet been established [115].

Metabolic reprogramming is also important for innate-like T lymphocytes. The development and functional specialization of mucosal-associated invariant T (MAIT) cells, gamma delta (γδ) T cells, and invariant natural killer T (iNKT) cells depend on the rates of glycolysis and fatty acid oxidation, mitochondrial status, and amino acid availability. Dependence on fatty acid oxidation and HIF-1α-dependent glycolysis has been demonstrated for distinct MAIT cell populations. Their proliferation requires activity of the axis involving the MYC transcription factor and solute carrier family 7 member 5 (SLC7A5), while mTORC1 signaling contributes to the response to mycobacterial antigens [116,117,118,119,120].

Clinical and experimental findings indicate that these cells contribute to immune abnormalities in TB and COPD. In patients with COPD, reduced MAIT cell counts and signs of their activation were associated with hospitalizations, while tobacco smoke components altered the interaction between major histocompatibility complex class I-related protein 1 (MR1) and MAIT cells. An expansion of natural killer (NK)-like cluster of differentiation 8-positive (CD8^+^) γδ T cells was observed during persistent infection. In patients with TB, hyperglycemia suppressed the function of γδ T cells expressing Vγ9 and Vδ2 receptor chains. In an experimental model of COPD, by contrast, clonally expanded interleukin 17-producing γδ T (γδT17) cells were associated with less severe emphysematous lung damage. These studies did not specifically assess the effects of malnutrition or negative energy balance, however, so the significance of the observed cellular changes in catabolic states remains unclear [121,122,123,124,125].

A possible causal role for mitochondrial dysfunction in lymphocytes has also been demonstrated experimentally. Transfer of exogenous mitochondria into cluster of differentiation 4-positive (CD4^+^) T cells reduced signs of cellular exhaustion and senescence and improved control of mycobacterial infection in animals. This experimental approach does not yet have a direct clinical application in TB or COPD. Nevertheless, the findings show that mitochondrial dysfunction may precede a decline in lymphocyte effector activity rather than arising solely as a consequence of prolonged activation [126].

### 4.4. Mitochondrial Dysfunction and Oxidative Stress

Mitochondria meet the energy requirements of skeletal muscles during exercise and recovery. Quadriceps biopsy specimens from patients with COPD have shown reduced oxygen consumption during succinate oxidation and reduced reserve capacity of cytochrome c oxidase. These changes indicate impaired oxidative phosphorylation (OXPHOS) [127]. As a result, anaerobic energy production may be recruited earlier, accompanied by lactate accumulation, early fatigability, and a further decline in physical activity.

At physiological levels, ROS contribute to muscle adaptation to exercise. Excessive ROS production or weakened antioxidant defenses, however, leads to oxidative damage to lipids, proteins, and nucleic acids. In mitochondria isolated from the vastus lateralis of 16 patients with COPD and 14 control participants, H_2_O_2_ production during active phosphorylation was almost threefold higher in the COPD group at comparable oxygen consumption: 147 ± 39 versus 51 ± 8 pmol∙mg^−1^∙h^−1^ (*p* < 0.001). Use of respiratory chain inhibitors identified complex III as the main source of ROS. The rate of H_2_O_2_ production correlated with the extent of oxidative damage to muscle tissue after exercise [128]. However, the control group consisted of patients with normal lung function undergoing surgery for lung cancer rather than healthy volunteers. This limits the interpretation of the comparison.

Another mechanism of injury is loss of mitochondrial DNA (mtDNA) integrity. In the vastus lateralis of 29 patients with COPD, oxidative DNA damage, mtDNA deletions, and cytochrome c oxidase-negative/succinate dehydrogenase-positive muscle fibers (COX^−^/SDH^+^) were more frequently detected than in 19 age-matched healthy participants [129]. The activity of genes responsible for the formation of new mitochondria could increase, but this was not followed by a corresponding increase in mitochondrial transcription factor A (TFAM) protein content or mtDNA copy number in the affected fibers. These changes indicate impaired coordination of the axis involving peroxisome proliferator-activated receptor gamma coactivator 1 alpha (PGC-1α), nuclear respiratory factor 1 (NRF1), and TFAM: activation of mitochondrial biogenesis genes is not accompanied by full mitochondrial recovery.

Inflammation and mitochondrial dysfunction may form a self-perpetuating vicious cycle. Activation of the canonical NF-κB pathway in muscle cells suppresses factors that maintain oxidative metabolism and reduces the production of OXPHOS components [89]. Damaged mitochondria, in turn, become a source of ROS that activate NF-κB, forkhead box O (FOXO) transcription factors, and protein breakdown pathways. When Parkin-dependent mitophagy is impaired, the cell cannot remove these mitochondria promptly. In an experimental study, exposure to cigarette smoke extract induced myotube atrophy and reduced Parkin expression by approximately 30%. Accumulation of mitochondrial ROS triggered MuRF1-dependent degradation of myosin heavy chains. In mice lacking the *Prkn* gene, prolonged smoke exposure reduced limb muscle mass by 31.7%. Similar changes were also detected in muscle tissue samples from patients with COPD and sarcopenia [75].

In TB, the effects of infection on mitochondria have been studied most extensively in macrophages. Tuberculosis necrotizing toxin degrades oxidized nicotinamide adenine dinucleotide (NAD^+^), leading to opening of the mitochondrial permeability transition pore, ROS accumulation, and death of the infected cell. Replenishment of NAD^+^ stores with nicotinamide or treatment with N-acetylcysteine reduced ROS production, protected macrophages, and restricted mycobacterial replication [130]. These processes may sustain systemic inflammation, but direct evidence of similar damage to skeletal muscle mitochondria in TB or coexisting TB and COPD is not yet available.

Low physical activity also contributes to a reduction in mitochondrial number but does not, by itself, explain all observed abnormalities. In a comparison of 12 patients with COPD and 21 healthy participants with similarly low levels of physical activity, lower OXPHOS measures, increased nonphosphorylating respiration, and more pronounced oxidative mitochondrial damage persisted in patients with COPD. Exercise training improved OXPHOS efficiency and reduced lipid peroxidation but did not fully restore maximal mitochondrial respiratory capacity or mitochondrial adaptation to exercise [131]. Deconditioning therefore exacerbates mitochondrial abnormalities but acts alongside mechanisms directly related to COPD.

Current evidence shows that the role of mitochondria in muscle abnormalities extends beyond inadequate ATP production and increased generation of reactive oxygen species. Defects in the mechanisms that maintain and renew functionally intact mitochondria have been described in COPD. In addition, sustained activation of the aryl hydrocarbon receptor (AhR) by tobacco smoke components may directly impair mitochondrial function in skeletal muscle. In experimental studies, the mitochondrial signaling peptides MOTS-c (mitochondrial open reading frame of the 12S ribosomal ribonucleic acid-c) and humanin attenuated dexamethasone-induced atrophy in human myotubes, while MOTS-c partially prevented muscle tissue loss in a cachexia model. These mechanisms have not yet been investigated in coexisting TB and COPD [132,133,134,135].

An adequate cellular supply of NAD^+^ is an important requirement for normal mitochondrial function. In a small randomized trial, nicotinamide riboside treatment in patients with COPD more than doubled whole-blood NAD^+^ levels and reduced sputum interleukin 8 (IL-8) concentrations; muscle NAD^+^ content and skeletal muscle function were not assessed [136]. However, a systematic review of studies of NAD^+^ precursors found no convincing improvement in muscle mass or function in older adults who had not been selected on the basis of baseline evidence of mitochondrial dysfunction [137]. Thus, an increase in blood NAD^+^ alone does not demonstrate recovery of muscle tissue; these studies provide no evidence of such an effect in TB–COPD.

Damaged mitochondria may not only lose their capacity to produce energy but also become a source of cellular danger signals. Release of mitochondrial DNA (mtDNA) into the cytoplasm activates signaling through cyclic guanosine monophosphate–adenosine monophosphate synthase (cGAS) and stimulator of interferon genes (STING) and may promote activation of the nucleotide-binding oligomerization domain, leucine-rich repeat, and pyrin domain-containing protein 3 (NLRP3) inflammasome. During tobacco smoke exposure, these mechanisms were associated with epithelial cell ferroptosis and, in the bronchial epithelium of patients with COPD, with interferon-dependent cellular senescence. In TB, the combination of lipid peroxidation and the type I interferon response sustained a damaging pattern of macrophage activation. However, these components were studied in different experimental and clinical models and cannot yet be regarded as a single established mechanism in coexisting TB and COPD [138,139,140,141,142].

Extracellular vesicles represent one possible route of signaling from the lungs to muscles: in an experimental study, exosomes from alveolar epithelial cells exposed to tobacco smoke impaired myotube differentiation and muscle function [143]. Such signaling has not been demonstrated in TB–COPD.

### 4.5. Impaired Skeletal Muscle Proteostasis

Skeletal muscle mass depends on the balance between protein synthesis and breakdown. One of the major anabolic regulatory pathways involves insulin-like growth factor 1 (IGF-1), phosphoinositide 3-kinase (PI3K), protein kinase B (Akt), and mTORC1. Activation of mTORC1 and its targets 70-kDa ribosomal protein S6 kinase (p70S6K) and eukaryotic translation initiation factor 4E-binding protein 1 (4E-BP1) enhances protein synthesis, whereas Akt phosphorylates FOXO transcription factors and prevents their translocation to the nucleus. When Akt activity decreases, forkhead box O1 (FOXO1) and forkhead box O3 (FOXO3) enter the nucleus and initiate the expression of genes involved in proteolysis and autophagy. Experimental evidence shows that the IGF-1/PI3K/Akt pathway not only stimulates protein synthesis but also suppresses catabolic processes, protecting muscle against glucocorticoid- or denervation-induced atrophy [76].

An attenuated anabolic response to feeding has been demonstrated at the level of whole-body protein metabolism in TB [51]. Alterations in Akt–mTORC1 and FOXO pathways have been described in muscle tissue in COPD [105,106]. These findings support consideration of anabolic resistance as a possible shared mechanism but do not establish a common postreceptor defect in IGF-1 signaling in patients with TB–COPD.

The ubiquitin–proteasome system participates in muscle protein breakdown. Substrate selection for ubiquitin-dependent proteasomal degradation is mediated by E3 ubiquitin ligases, including the muscle ligases muscle atrophy F-box protein (atrogin-1/MAFbx), encoded by the F-box protein 32 gene (*FBXO32*), and MuRF1, encoded by the tripartite motif-containing 63 gene (*TRIM63*). In experimental models, FOXO factors stimulated the expression of both ligases [76], whereas activation of the IKKβ/NF-κB pathway in mice caused atrophy in which increased MuRF1 expression played an important role [90]. Differences between muscles within the same patients have been identified in COPD. In 12 patients with COPD, but not in 7 control participants, *FBXO32*, *TRIM63*, and *FOXO1* messenger ribonucleic acid (mRNA) expression was higher in the quadriceps than in the diaphragm. Phosphorylated p70S6K content was also lower in the quadriceps [106]. These differences are consistent with the involvement of local factors, including the pattern of muscle loading, in the regulation of muscle mass, although the study does not establish a direct causal effect of the level of loading.

Autophagy is another important component of proteostasis. It removes protein aggregates and damaged organelles and is therefore not inherently pathological. Increased autophagosome numbers, enhanced microtubule-associated protein 1 light chain 3 beta (LC3B) lipidation, and increased expression of autophagy genes have been detected in muscle tissue from patients with COPD. These changes were accompanied by AMPK and FOXO activation and reduced Akt–mTORC1 pathway activity [105]. A single measurement of the lipidated form of LC3B (LC3B-II) or autophagosome number cannot distinguish increased autophagic flux from impaired lysosomal degradation. Autophagy may therefore represent either a protective response that removes damaged structures or a component of excessive muscle tissue breakdown.

The extent of oxidative damage does not fully explain differences in muscle mass in COPD. In severe COPD, oxidative damage to muscle proteins was increased regardless of body composition. Even in patients without muscle wasting, myosin heavy chains were more extensively oxidized than in healthy participants, although their abundance was preserved. Marked protein ubiquitination, increased levels of atrogin-1, FOXO1, and the NF-κB p65 subunit, and loss of myosin were observed only in patients with muscle wasting [144].

Myostatin and activins form a distinct regulatory system. Myostatin belongs to the transforming growth factor beta (TGF-β) family and activates SMAD family members 2 and 3 (SMAD2/3) by binding to activin type II receptors. This limits muscle fiber differentiation and growth, suppresses the Akt–mTOR pathway, and increases the expression of atrophy-related genes. Serum myostatin concentrations were higher in 71 patients with stable COPD than in 60 age-matched healthy individuals: 11.85 ± 4.01 versus 7.46 ± 2.21 ng/mL (*p* < 0.01). Muscle mass was reduced in both men and women, but its inverse association with myostatin levels was detected only in men [145]. In a study involving 23 patients with COPD and sarcopenia, 18 patients without sarcopenia, and 13 healthy individuals, vastus lateralis biopsy specimens showed evidence of myostatin–SMAD2/3 pathway activation in sarcopenia, including increased myostatin protein content and higher phosphorylated-to-total SMAD2 and SMAD3 ratios. In patients with COPD and sarcopenia, muscle myostatin content and the p-SMAD3/SMAD3 ratio were negatively correlated with fat-free mass index: r = −0.727 (*p* = 0.026) and r = −0.703 (*p* = 0.035), respectively [146]. A trial of bimagrumab, which blocks activin type II receptors, in patients with COPD and low muscle mass is discussed in Section 7.6 [147].

The studies reviewed provide direct evidence of impaired protein metabolism and an attenuated anabolic response to feeding in active TB [51], whereas tissue studies of the proteasome system, autophagy, and myostatin signaling have been conducted predominantly in COPD [105,106,144,145,146]. Their findings do not establish the nature of concurrent abnormalities in these systems when the diseases coexist.

mTORC1 activity depends on the availability of several amino acids, not only leucine. The kinase general control nonderepressible 2 (GCN2) senses amino acid deficiency, including insufficient glutamine and asparagine, and increases the expression of regulated in development and DNA damage responses 1 (REDD1) and Sestrin2 through activating transcription factor 4 (ATF4); both proteins suppress mTORC1 activity. In an experimental model of cancer cachexia, disruption of the methionine cycle and reduced S-adenosylmethionine content were accompanied by hypomethylation of the *Ddit4* gene and increased expression of its encoded protein, REDD1. These mechanisms have not been directly investigated in TB, but they point to molecular processes that may contribute to anabolic resistance and are amenable to experimental evaluation [148,149].

In addition to atrogin-1 and MuRF1, E3 ubiquitin ligases containing a UBR domain participate in protein quality control. Single-nucleus studies have shown that the magnitude and direction of catabolic programs differ among myonuclear populations and other cells within muscle tissue. Reduced muscle function in sarcopenia is associated not only with muscle mass loss but also with impaired neuromuscular transmission and altered transcriptional programs in specific muscle fiber types. Experimental findings also show that sustained interferon signaling and inflammation-induced epigenetic changes may inhibit the formation of new muscle fibers, accelerate cellular senescence, and increase the susceptibility of muscle progenitor cells to ferroptosis [83,150,151,152,153,154].

The muscle-secreted Dickkopf-related protein 3 (DKK3) was studied in two clinical cohorts of patients with COPD and sarcopenia. Its circulating levels and expression in muscle tissue were associated with muscle mass and oxidative stress measures. These findings support consideration of DKK3 as a potential research marker of muscle abnormalities in COPD. However, its specificity for coexisting TB and COPD and its ability to predict subsequent changes in muscle mass and function have not yet been established [155].

### 4.6. Adipose Tissue and Endocrine Regulation

Changes in adipose tissue are accompanied by alterations in adipokine regulation. In active TB, reduced leptin levels and changes in other appetite regulators are associated with the size of fat stores and the course of the disease [50,52]. Adipokine concentrations therefore cannot be interpreted without body composition data; the sequence of fat and muscle tissue loss varies among patients [19].

Adiponectin increases tissue insulin sensitivity; however, an elevated concentration in COPD does not always indicate favorable metabolic status. It may be associated with reduced fat mass and a predominantly emphysematous phenotype. In a cohort of 196 patients with COPD, the associations of leptin and adiponectin levels and their ratio with disease severity and progression varied according to clinical phenotype [156]. This indicates heterogeneity in the alterations in adipokine regulation in COPD.

In active TB, increased resistin levels and altered concentrations of ghrelin and peptide YY have been reported alongside reduced appetite and fat mass. Some of these abnormalities resolve during anti-tuberculosis treatment [50]. Elevated ghrelin levels in patients with wasting may represent a compensatory response to reduced food intake or indicate decreased sensitivity to this hormone. A high ghrelin concentration therefore does not necessarily mean that its effect on appetite is preserved. A multicenter randomized trial enrolled 33 patients with COPD and cachexia. During pulmonary rehabilitation, they received intravenous ghrelin at a dose of 2 μg/kg twice daily for three weeks. By the end of week 3, the within-group increase in six-minute walk distance was 40 m (*p* = 0.033), and the improvement was maintained at seven weeks. However, the between-group difference in walking distance compared with placebo did not reach statistical significance [157].

Inflammation also affects the hypothalamic–pituitary–adrenal and gonadal axes. Men with newly diagnosed, untreated pulmonary TB had markedly reduced dehydroepiandrosterone and testosterone levels, moderately increased cortisol and estradiol concentrations, and elevated growth hormone levels. These changes occurred alongside increased IL-6, interleukin 10 (IL-10), and IFN-γ levels. Taken together, this hormonal profile is consistent with the shift toward a hypercatabolic state described by the authors [77].

The dependence of adipokine levels on body composition and the variable associations between hormones and clinical status limit their diagnostic use [50,77,156]. Diagnostic thresholds and the incremental value of multihormone panels for detecting muscle depletion in TB–COPD have not been established.

Adipose tissue may contribute to TB pathogenesis beyond its role as a source of energy and adipokines. *M. tuberculosis* entered cultured human visceral white adipocytes. Intracellular mycobacteria expressed genes characteristic of dormancy, whereas infected adipocytes increased their production of proinflammatory mediators. In experimental models, both adipose tissue loss and excessive accumulation altered pulmonary immune responses, with the nature of these changes depending on the sex of the animals. However, these studies were conducted outside the clinical context of coexisting TB and COPD; their findings therefore do not establish adipose tissue as a reservoir of *M. tuberculosis* in patients [158,159].

In cachexia, fat mass loss may result not only from increased lipolysis but also from greater energy expenditure on heat production. In an experimental model of cancer cachexia, the glucose-regulated protein 75 (GRP75) promoted the conversion of white adipose tissue to a thermogenically active state, a process known as browning. The contribution of this mechanism to fat mass loss in TB has not yet been demonstrated. In a longitudinal study of individuals exposed to tobacco smoke, the development of low muscle mass was associated with more severe lung disease and lower leptin concentrations. Because leptin and the melanocortin system are involved in both appetite regulation and immune defense against infection, leptin levels should be assessed in the context of the amount and distribution of adipose tissue [160,161,162].

### 4.7. Ceramides and Sphingolipid Metabolism

Ceramides are bioactive sphingolipids involved in the regulation of muscle protein synthesis and breakdown [163]. In L6 myotubes, exogenous C2-ceramide reduced the abundance of the sodium-coupled neutral amino acid transporter 2 (SNAT2) at the plasma membrane, decreased the intracellular amino acid pool, and suppressed protein synthesis [164]. In another study, exposure of differentiated L6 myotubes to TNF-α was accompanied by atrophy and an approximately 35% increase in total ceramide content relative to controls [163]. Pharmacological inhibition of ceramide formation in this model reduced proteolysis and *Fbxo32* and *Map1l*c*3b* gene expression while increasing protein synthesis [163]. In mice bearing C26 carcinoma, ceramide content in the tibialis anterior muscle was 53% higher than in animals without tumors. Myriocin administration partially attenuated atrophy: the mass of this muscle was 11% higher than in untreated tumor-bearing animals (*p* = 0.05) [163].

Additional evidence comes from studies of muscle aging: inhibition of serine palmitoyltransferase, the enzyme catalyzing the initial step of de novo sphingolipid synthesis, improved proteostasis and mitochondrial function in cultured human myoblasts and the skeletal muscles of aging mice [165]. However, the effect depended on the intervention target: inhibition of ceramide synthase 1 (CERS1), by contrast, exacerbated age-related declines in muscle mass and function in mice [166]. Ceramide species composition is also relevant: in a study involving nine young and ten older men, muscle C16:0- and C20:0-ceramide levels were 156% and 30% higher, respectively, in older participants (*p* < 0.05 for both comparisons), whereas no difference in total ceramide content was detected [167]. C16:0-ceramide content was inversely correlated with lower-leg fat-free mass (*p* = 0.008), but this association does not establish a causal role for ceramides in muscle wasting [167]. Thus, these findings support further investigation of sphingolipid metabolism but do not, by themselves, establish the efficacy of its pharmacological modulation for muscle wasting in patients with TB or COPD [163,165,166].

Ceramides may contribute to the metabolic effects of tobacco smoke: conditioned medium from lung cells exposed to smoke extract suppressed the insulin response and mitochondrial respiration in C2C12 myotubes, whereas myriocin attenuated these abnormalities [168]. High-mobility group box 1 (HMGB1) is a potential mediator: its levels increased in smoke-exposed mice, and, in separate experiments, HMGB1 administration induced muscle ceramide accumulation and reduced insulin sensitivity and mitochondrial respiration; myriocin exerted a protective effect [169]. However, when smoke exposure was combined with a high-fat, high-sugar diet, myriocin improved muscle mitochondrial respiration but did not prevent insulin resistance [168]. More recent studies have also demonstrated direct inhibition of respiration in isolated muscle mitochondria by smoke constituents, including nicotine and o-cresol [170]. The partial restoration of muscle fiber respiration after smoke condensate exposure by the mitochondria-targeted antioxidant MitoTEMPO further implicates oxidative stress [171].

A study of lung tissue in COPD (69 patients) found reduced sphingosine kinase 1 activity and an absence of the correlation between ceramide and sphingosine-1-phosphate levels observed in smokers without COPD [172]. Local ceramide content correlated with active caspase-3, a marker of apoptosis; however, total ceramide content was not elevated in the COPD group overall and differed across disease stages [172]. Higher levels of individual plasma ceramides, particularly C18:0, were associated with lower FEV_1_ in a Korean sample and the population-based Cardiovascular Health Study cohort (CHS; 2675 participants) [173,174]. In a longitudinal analysis of the same CHS cohort involving 4612 participants, elevated plasma levels of ceramides with 16:0 and 18:0 fatty-acyl chains (Cer-16 and Cer-18, respectively) were associated with a higher risk of death from chronic respiratory diseases [175]. These observational findings do not establish causality or permit conclusions about ceramide accumulation in the skeletal muscles of patients with COPD [172,174,175].

During mycobacterial infection, sphingolipid metabolism contributes to interactions between the pathogen and the host cell. The *M. tuberculosis* protein Rv0888 hydrolyzes sphingomyelin to produce ceramide and phosphocholine, which the bacterium uses as nutrient substrates; its function has been investigated biochemically and in macrophage cultures [176]. In another study, myriocin reduced the bacterial burden in infected cells and mice, but its antimycobacterial effect was linked to the lipid droplet protein perilipin 2 (PLIN2) and could not be attributed solely to ceramide reduction [177]. Thus, these studies do not provide a basis for using ceramide synthesis inhibitors to treat cachexia or sarcopenia in TB–COPD [163,168,176,177].

### 4.8. Factors Contributing to Persistent Muscle Depletion

Insufficient oxygen availability may contribute to reduced oxidative metabolism. In cellular models, hypoxia activated HIF-1-dependent expression of pyruvate dehydrogenase kinase 1 (PDK1), limiting pyruvate entry into the tricarboxylic acid cycle and sustaining ATP production through glycolysis [178]. In cultured muscle cells, hypoxia also suppressed the peroxisome proliferator-activated receptor (PPAR)/PGC-1α pathway and the expression of mitochondrial components through HIF-1α [179]. These findings characterize cellular adaptation to hypoxia but do not establish that this mechanism sustains muscle depletion in patients with TB–COPD. In the clinical setting, dyspnea and hyperinflation should also be considered: they increase the work of breathing, may make eating difficult, and limit daily activity [3].

Low physical activity is accompanied by deconditioning, and muscle studies in COPD indicate the importance of exercise for anabolic signaling and mitochondrial adaptation [38,106,131]. In a three-year follow-up of 137 patients with COPD and 26 patients with chronic bronchitis, step count declined by an average of 393 per year; reduced activity was associated with deterioration in FEV_1_ and health status [180].

Tobacco smoke exposure can also damage skeletal muscle. Muscle biopsies from patients with severe and very severe COPD showed fiber-type grouping consistent with repeated cycles of denervation and reinnervation. A low fat-free mass index was accompanied by greater accumulation of small fibers expressing markers of persistent denervation and reduced expression of genes involved in restoring innervation [181]. In a separate part of the same study, 16 weeks of passive tobacco smoke exposure in mice caused neuromuscular junction degeneration before the emergence of a fiber-type shift and atrophy [181]. Thus, a causal effect of smoke was demonstrated experimentally, whereas the human data describe associations in COPD; coexisting TB–COPD was not investigated in this study. Other cellular and animal experiments link smoke exposure to insufficient Parkin-dependent mitophagy, accumulation of damaged mitochondria, and increased oxidative stress [75].

During exacerbations and hospitalizations, muscle is simultaneously affected by inflammation, hypoxemia, inactivity, and deteriorating nutritional intake. Among 55 patients hospitalized for an exacerbation of chronic respiratory disease, quadriceps thickness decreased by an average of 8.3%. The loss persisted at six weeks, and recovery was only partial at three months. Readmissions had a particularly adverse effect on recovery [66]. When such episodes recur, incomplete recovery after each episode gradually reduces muscle and functional reserve. Because hospitalizations and, in some studies, exacerbations are more frequent in patients with COPD after previous TB than in other patients with COPD [5,15,33,34], this mechanism may be particularly relevant to this group.

Systemic glucocorticoids are used for COPD exacerbations according to established indications [3]. Repeated or prolonged exposure may increase muscle catabolism. In experimental models, glucocorticoid-induced atrophy is associated with attenuated Akt–mTOR signaling and activation of muscle catabolism genes [76]. In an experiment using *Ddit4*-knockout mice, seven days of dexamethasone administration did not cause the reductions in the mass of the muscles studied and in muscle fiber area that were observed in wild-type mice; protein synthesis and mTORC1 activity were also preserved after a single dose [182]. These experimental findings explain a possible mechanism of steroid myopathy but do not establish the risk associated with standard short courses for COPD exacerbations.

Age must be considered when interpreting muscle abnormalities: muscle strength and the capacity for recovery decline with age, while the likelihood of sarcopenia increases [26]. Consequently, in an older patient, the sequelae of TB and the effects of COPD may be superimposed on preexisting muscle depletion. Studies with age-matched groups and repeated measurements are needed to distinguish the contributions of age, infection, airflow obstruction, hypoxemia, and low physical activity.

**Figure 2:**
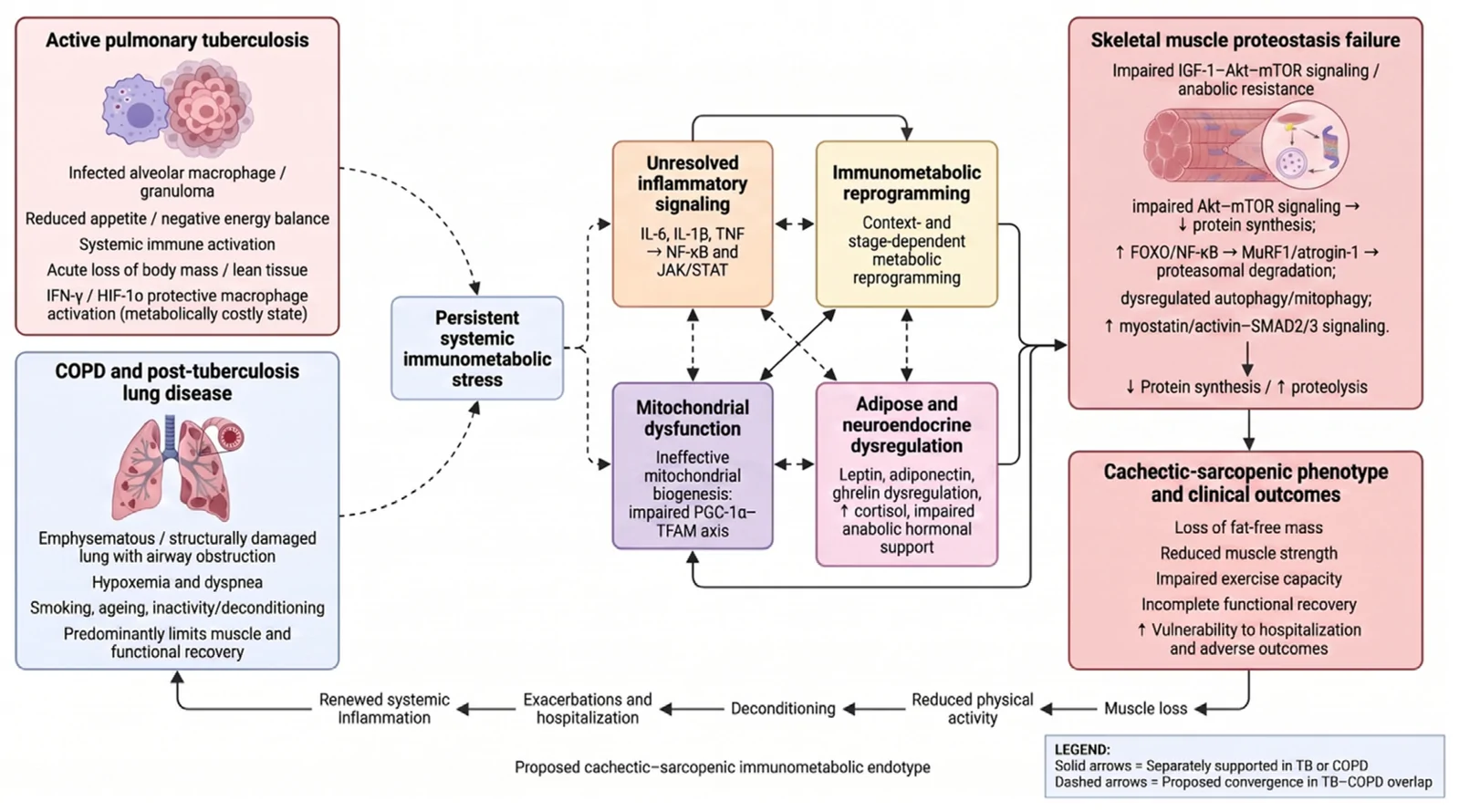
Immunometabolic mechanisms linking tuberculosis and COPD to muscle wasting. Active tuberculosis (TB), COPD, and post-tuberculosis lung disease sustain systemic immunometabolic stress characterized by persistent inflammation, metabolic reprogramming, mitochondrial dysfunction, and disturbances in adipose tissue function and neuroendocrine regulation. Together, these processes disrupt skeletal muscle proteostasis by attenuating anabolic signaling and enhancing proteolysis, autophagy, and myostatin–activin signaling, leading to reduced fat-free mass, muscle weakness, impaired exercise tolerance, and incomplete functional recovery. Muscle wasting and reduced physical activity create a feedback loop that perpetuates deconditioning, exacerbations, and systemic inflammation. Solid arrows indicate links supported by evidence from TB or COPD individually, whereas dashed arrows indicate the hypothesized convergence of mechanisms when these conditions coexist. This schematic provides a conceptual synthesis and does not establish that the entire proposed causal sequence occurs in patients with coexisting TB and COPD. Created in BioRender. Oskin, D. (2026) https://BioRender.com/y090gu3. Abbreviations: Akt, protein kinase B; COPD, chronic obstructive pulmonary disease; FOXO, forkhead box O; HIF-1α, hypoxia-inducible factor 1 alpha; IFN-γ, interferon gamma; IGF-1, insulin-like growth factor 1; IL-1β, interleukin 1 beta; IL-6, interleukin 6; JAK, Janus kinase; mTOR, mechanistic target of rapamycin; MuRF1, muscle RING finger protein 1; NF-κB, nuclear factor kappa-light-chain-enhancer of activated B cells; PGC-1α, peroxisome proliferator-activated receptor gamma coactivator 1 alpha; SMAD2/3, SMAD family members 2 and 3; STAT, signal transducer and activator of transcription; TB, tuberculosis; TB–COPD, coexisting tuberculosis and chronic obstructive pulmonary disease; TFAM, mitochondrial transcription factor A; TNF, tumor necrosis factor.

The immune interactions described above are summarized in Figure 2.

## 5. Overview of the Mechanisms of Muscle Wasting

The processes discussed may jointly influence muscle mass and function, but the degree of clinical support for them varies. Table 3 compares the principal mechanisms, their proposed consequences, and the sources of evidence, including the data available in patients with TB–COPD. This distinction separates established clinical abnormalities from experimental hypotheses [51,75,76,77,163,168,172,176,177].

## 6. Diagnosis of Malnutrition and Muscle Impairment

### 6.1. Assessment of Nutrition and Changes in Body Weight

The initial assessment of nutritional status includes measuring height and body weight, calculating BMI, and assessing weight changes and dietary intake [70]. Assessment addresses unintentional weight loss, changes in appetite, symptoms that impair food intake or absorption, and access to adequate nutrition [28,70,183]. WHO recommends nutritional assessment and individualized dietary counseling for all patients with TB at diagnosis and throughout treatment. This is a strong recommendation, although direct evidence of the independent effects of these measures on TB outcomes is lacking [70].

Validated tools, such as the Malnutrition Universal Screening Tool (MUST) or Nutritional Risk Screening 2002 (NRS-2002), are used to identify the risk of malnutrition. Subsequent diagnosis follows the GLIM criteria, which require at least one phenotypic and one etiologic criterion [27,28]. Phenotypic criteria include unintentional weight loss of >5% within the past 6 months or >10% over a period longer than 6 months, BMI < 20 kg/m^2^ in individuals younger than 70 years or <22 kg/m^2^ in those aged 70 years or older, and low muscle mass. For Asian populations, the corresponding BMI thresholds are <18.5 and <20 kg/m^2^ [28]. Etiologic criteria comprise reduced food intake or assimilation and disease-related inflammation [28]. In the 2025 GLIM update, the criterion for reduced intake is met by an energy intake of ≤50% of requirements for more than one week or any reduction in food intake lasting more than two weeks [28].

In active TB, the GLIM inflammation criterion may be established on clinical grounds; CRP is measured additionally when the clinical presentation is inconclusive [184]. Muscle mass is assessed using a validated method and cutoffs appropriate for sex, population, and measurement technique [185]. Edema and severe obesity may reduce the accuracy of this assessment [28]. Handgrip dynamometry measures muscle strength but does not replace the assessment of muscle mass in the GLIM diagnosis of malnutrition [28,185].

The European Respiratory Society (ERS) statement proposes a combination of unintentional weight loss of >5% over 6 months and a fat-free mass index (FFMI) <17 kg/m^2^ in men or <15 kg/m^2^ in women to identify cachexia in COPD [186]. These FFMI cutoffs were proposed primarily for patients with normal or low body weight [186]. Because unintentional weight loss was independently associated with mortality in a prospective cohort of patients with stable COPD (Section 3.5), regular weight monitoring is needed also in patients with preserved FFMI [10]. However, this study does not establish the validity of these criteria in patients with coexisting COPD and active TB [10].

### 6.2. Assessment of Muscle Strength and Mass

According to EWGSOP2 recommendations, the SARC-F questionnaire, which assesses strength, assistance with walking, rising from a chair, climbing stairs, and falls, is used for initial identification of sarcopenia risk. When sarcopenia is clinically suspected, muscle strength is assessed regardless of the questionnaire result [26]. In a study of 205 patients with COPD referred for pulmonary rehabilitation, SARC-F sensitivity was only 13%, confirming the limitations of this screening approach in this population [187]. Handgrip strength <27 kg in men and <16 kg in women, or a time of >15 s to complete five chair rises without using the arms, indicates probable sarcopenia [26]. In Asian populations, AWGS 2019 uses higher strength cutoffs (<28 kg in men and <18 kg in women) and a time of ≥12 s for five chair stands to define possible sarcopenia in primary care [29]. For adults aged 50–64 years, AWGS 2025 sets the handgrip strength cutoffs at <34 kg in men and <20 kg in women [30].

The diagnosis is confirmed when low strength is accompanied by reduced muscle mass or quality, although clinical methods for assessing muscle quality have not yet been standardized. For DXA measurements, the appendicular muscle mass index cutoffs are <7.0 kg/m^2^ in men and <5.5 kg/m^2^ in women [26]. Additional impairment of physical performance, defined as usual gait speed ≤0.8 m/s or a Short Physical Performance Battery (SPPB) score ≤8, indicates severe sarcopenia. EWGSOP2 cutoffs are intended for European populations; in young patients with TB, they should be interpreted in light of available age-specific and population-specific reference values [26]. For Asian patients, AWGS 2019 defines low appendicular muscle mass as <7.0 kg/m^2^ in men and <5.4 kg/m^2^ in women by DXA, or <7.0 and <5.7 kg/m^2^ by bioimpedance [29]. For adults aged 50–64 years, AWGS 2025 sets the height-adjusted appendicular muscle mass index cutoffs at <7.2 kg/m^2^ in men and <5.5 kg/m^2^ in women by DXA and at <7.6 and <5.7 kg/m^2^ by bioimpedance, and it retains the AWGS 2019 cutoffs for handgrip strength and muscle mass in adults aged 65 years or older [30]. Because AWGS 2025 provides thresholds only for adults aged 50–64 and ≥65 years, these age-specific cutoffs should not be applied automatically to patients younger than 50 years, in whom age-appropriate reference values are needed.

The six-minute walk test assesses overall exercise tolerance, while concurrent pulse oximetry can detect exertional desaturation [6]. A reduced walking distance is not specific to sarcopenia because exercise tolerance depends on respiratory, cardiovascular, and muscular limitations [188]. Body weight and composition, muscle strength, and exercise tolerance should therefore be assessed separately during follow-up [6,26,188].

### 6.3. Pulmonary Function Testing and Distinction Between Post-Tuberculosis Airflow Obstruction and Chronic Obstructive Pulmonary Disease

After completion of TB treatment, assessment includes respiratory symptoms, chest radiography, spirometry with bronchodilator testing, and pulse oximetry. Additional functional tests are performed as clinically indicated [6]. CT is used to characterize bronchiectasis, residual cavities, and focal lesions when radiographic findings are insufficient to guide further management [6].

The spirometric criterion for COPD is a post-bronchodilator ratio of FEV_1_ to forced vital capacity (FVC) <0.70, but diagnosis requires interpretation alongside symptoms, medical history, and risk factors [3,189]. A history of TB and subsequent airflow obstruction are not, by themselves, sufficient to diagnose COPD. The nature of post-tuberculosis structural changes and other potential causes of airflow obstruction must be considered [6,189].

**Figure 3:**
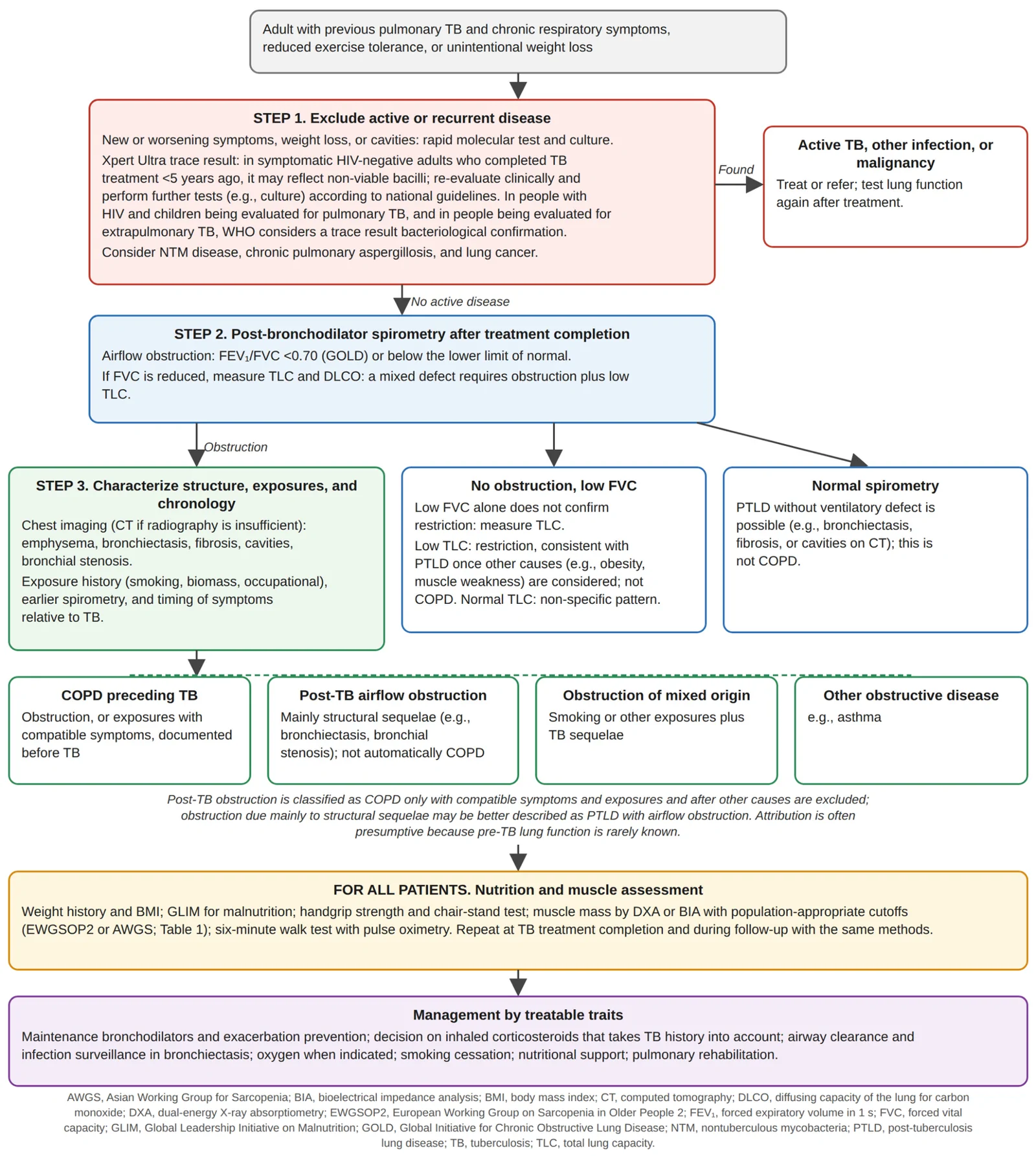
Proposed three-step approach to patients with previous pulmonary TB and chronic respiratory symptoms, reduced exercise tolerance, or unintentional weight loss. Step 1 excludes active or recurrent TB, other infections, and malignancy. An Xpert MTB/RIF Ultra trace result may reflect non-viable bacilli in symptomatic HIV-negative adults who completed TB treatment less than five years earlier; in people living with HIV and children being evaluated for pulmonary TB and in people being evaluated for extrapulmonary TB, WHO considers it bacteriological confirmation. Step 2 determines whether airflow obstruction is present by post-bronchodilator spirometry after completion of TB treatment; when FVC is reduced, total lung capacity and diffusing capacity are measured, because low FVC alone does not confirm restriction and a mixed defect requires both obstruction and a reduced total lung capacity. Step 3, in patients with obstruction, characterizes structural damage by chest imaging and considers exposure history, earlier lung function, and the timing of symptoms relative to TB. Post-tuberculosis airflow obstruction is classified as COPD only when it is accompanied by compatible symptoms and exposures and other causes are excluded; obstruction explained mainly by structural sequelae may be better described as post-tuberculosis lung disease with airflow obstruction, and post-tuberculosis lung disease without airflow obstruction is not COPD. Assessment of nutritional status and muscle mass and function is recommended for all patients, and management is then organized by treatable traits (Section 7.4). The approach is based on existing standards [3,6,189,193,194] and has not been validated. Colors indicate the purpose of each component: red, exclusion or management of active disease; blue, pulmonary function assessment and non-obstructive patterns; green, characterization of airflow obstruction; orange, nutritional and muscle assessment for all patients; and purple, management by treatable traits. Additional abbreviations: COPD, chronic obstructive pulmonary disease; FVC, forced vital capacity; HIV, human immunodeficiency virus; MTB, Mycobacterium tuberculosis; RIF, rifampicin; TB, tuberculosis; WHO, World Health Organization.

Because post-tuberculosis lung disease comprises obstructive, restrictive, and mixed ventilatory defects with various structural sequelae [6,190,191], spirometry alone does not distinguish post-tuberculosis airflow obstruction from COPD. Lung function may change during and after treatment, including a transition from a restrictive to an obstructive pattern, so the type of impairment should be confirmed after treatment completion [6,192]. A reduced FVC does not in itself confirm restriction, which requires measurement of total lung capacity, and diffusing capacity helps to characterize impaired gas exchange [193]. We propose a three-step approach (Figure 3) that is based on existing standards but has not been validated.

First, active or recurrent TB, other infections, and malignancy are excluded in patients with new or worsening symptoms, weight loss, or cavities, as described below [6,194]. Second, post-bronchodilator spirometry performed after treatment completion establishes whether airflow obstruction is present (FEV_1_/FVC < 0.70 or below the lower limit of normal) [3,189,193]; when FVC is reduced, total lung capacity and diffusing capacity are measured, because a mixed defect requires both obstruction and a reduced total lung capacity [193]. Third, in patients with airflow obstruction, chest imaging, including CT when radiography is insufficient, characterizes the structural substrate (emphysema, bronchiectasis, fibrosis, cavities, and bronchial stenosis). Exposure history (tobacco smoking, biomass, and occupational exposures), earlier spirometry, and the timing of respiratory symptoms relative to TB help to judge whether the obstruction preceded TB, developed after it, or has both origins [3,6,190,195]. On this basis, obstruction can be attributed to COPD that preceded TB, post-tuberculosis airflow obstruction due mainly to structural sequelae, obstruction of mixed origin (smoking or other exposures together with TB), or another obstructive disease such as asthma. In patients without obstruction, a reduced total lung capacity indicates restriction, which is consistent with post-tuberculosis lung disease once other causes have been considered; a reduced FVC with normal total lung capacity is a non-specific pattern; and structural sequelae with normal spirometry indicate post-tuberculosis lung disease without a ventilatory defect [193]. None of these is COPD. Post-tuberculosis airflow obstruction is not automatically COPD either: the diagnosis requires compatible symptoms and exposures and the exclusion of other causes, and obstruction explained mainly by bronchiectasis or bronchial stenosis may be better described as post-tuberculosis lung disease with airflow obstruction [3,6,195]. In practice, the attribution often remains presumptive because lung function before TB is rarely known [195].

This classification matters for the assessment of muscle wasting. Bronchiectasis, fibrosis, and cavities increase the risk of recurrent infection, exertional desaturation, and weight loss, and restrictive or mixed defects limit exercise tolerance independently of muscle function [6]. In all categories, assessment includes GLIM-based diagnosis of malnutrition, evaluation for sarcopenia with population-appropriate criteria (EWGSOP2 or AWGS), and a six-minute walk test with pulse oximetry [6,26,28,29].

Worsening cough, increasing dyspnea, or unintentional weight loss after TB treatment requires evaluation to exclude recurrent TB, nontuberculous mycobacterial disease, chronic pulmonary aspergillosis, and malignancy, particularly in the presence of residual cavities [6]. WHO-recommended rapid molecular tests are used initially when active TB is suspected [194]. In symptomatic HIV-negative adults being evaluated for pulmonary TB who successfully completed TB treatment less than five years earlier, an Xpert Ultra trace result, and occasionally low or very low results, may reflect persistent DNA from nonviable mycobacteria [194]. These results require clinical and radiological assessment and additional investigations, including liquid culture, and do not exclude active TB. A trace result is considered bacteriological confirmation of TB in people living with HIV and children being evaluated for pulmonary TB, as well as in people being evaluated for extrapulmonary TB [194].

### 6.4. Laboratory Investigations

Laboratory investigations aim to identify comorbid and potentially reversible causes of deterioration, including anemia, disorders of glucose metabolism, HIV infection, and liver and kidney disease. The specific tests are selected according to clinical findings [6,70]. Hyperglycemia may be transient during active infection; conditions affecting erythrocyte lifespan must be considered when interpreting HbA1c [42,43,44]. Albumin and prealbumin reflect inflammatory burden and the risk of adverse outcomes but are not direct measures of protein stores or muscle mass [196].

Cytokines, myostatin, GDF-15, ceramides, and measures of mitochondrial metabolism remain research parameters and are not included in the standard diagnostic assessment of malnutrition or sarcopenia. Clinical follow-up uses repeated measurements of body weight, muscle strength, and physical performance obtained with comparable methods [6,26,28,70].

## 7. Treatment and Rehabilitation

Correction of malnutrition and restoration of physical performance are undertaken alongside treatment for TB and COPD. The components and intensity of interventions are determined by nutritional status, functional limitations, and clinical stability. In patients with coexisting disease, it must be recognized that evidence for the effectiveness of these interventions comes predominantly from studies of TB or COPD separately [70,186,188].

### 7.1. Nutritional Support in Active Tuberculosis

WHO recommends nutritional interventions for all patients with TB and malnutrition, irrespective of its severity. This is a strong recommendation based on low- to moderate-certainty evidence [70]. The recommendation is based on a review of 17 randomized controlled trials (RCTs), although findings for individual interventions come from small samples. High-energy, protein-rich nutritional support may increase the likelihood of treatment completion (one RCT, n = 100; RR 1.20; 95% CI 1.04–1.37; low certainty) and probably improves handgrip strength at three months (two RCTs, n = 136; mean difference 1.51 kg; 95% CI 1.10–1.92; moderate certainty). The form of support is selected according to actual food intake, nutritional deficits, tolerability, and food availability [70].

In an open-label RCT by Kumar et al., 335 adults with drug-susceptible pulmonary TB and BMI 14–18.5 kg/m^2^ were assigned to a high-energy nutritional supplement plus dietary advice or dietary advice alone. Participants in the intervention group were more likely to gain ≥5% of baseline body weight at two months (53.9% vs. 39.3%) and ≥10% at six months (55.8% vs. 41.0%) [71]. Six patients were excluded after randomization because drug resistance was detected. Gastrointestinal symptoms were more common with supplementation but did not require modification of the intervention. These findings indicate improved weight recovery but do not establish an effect of the supplement on muscle mass, strength, or mortality [71].

Vitamin and mineral supplements are prescribed to correct documented deficiencies or for other indications, rather than as a stand-alone intervention to improve TB outcomes [70]. In patients with severe wasting or prolonged inadequate food intake, the risk of refeeding syndrome is assessed before nutritional support is initiated. High-risk patients receive thiamine before feeding, with monitoring of phosphate, potassium, and magnesium concentrations and fluid balance. The initial energy intake and rate of increase are individualized according to electrolyte abnormalities and the feasibility of monitoring [197,198]. When baseline phosphate, potassium, or magnesium concentrations are severely reduced, the American Society for Parenteral and Enteral Nutrition (ASPEN) recommends delaying the initiation of feeding or an increase in energy intake until these abnormalities have been corrected [197]. These measures are based on general consensus recommendations rather than studies in patients with coexisting TB and COPD [197,198].

### 7.2. Nutritional Interventions in Chronic Obstructive Pulmonary Disease

In COPD, nutritional care is tailored to the severity of malnutrition and rehabilitation goals; no single formulation has been established for all patients [186]. In a double-blind RCT by Calder et al., 45 patients with COPD and weight loss or low BMI received a formulation containing protein, omega-3 fatty acids, and vitamin D or an isocaloric comparator for 12 weeks. The primary objective was to assess safety, and adverse event profiles were comparable. The formulation produced a greater increase in fat mass and improvements in selected secondary post-exercise outcomes, but no advantage in appendicular lean mass [72].

In a randomized trial of targeted nutritional supplementation during exercise training, 81 patients with COPD and low muscle mass received a supplement containing leucine, vitamin D, and omega-3 fatty acids or placebo during a four-month exercise program. Muscle mass, quadriceps strength, and endurance improved in both groups. The supplement increased body weight but provided no additional benefit for muscle mass or quadriceps strength, the primary endpoint. Baseline protein intake was adequate, limiting the applicability of these findings to patients with severe wasting [73].

The response to nutritional support is therefore assessed through changes in food intake and body weight alongside body composition, strength, and physical performance. When weight gain is predominantly attributable to fat mass, muscle recovery requires continued assessment [18,72,73,186].

### 7.3. Pulmonary Rehabilitation

The American Thoracic Society (ATS) recommends pulmonary rehabilitation for adults with stable COPD and after hospitalization for an exacerbation. Both recommendations are strong and supported by moderate-certainty evidence [188]. Programs include individualized aerobic and resistance training, patient education, and support for regular physical activity; they improve exercise tolerance, reduce dyspnea, and enhance quality of life. In a meta-analysis of 12 RCTs (n = 1309), rehabilitation initiated within three weeks after discharge reduced the odds of readmission compared with usual care (odds ratio 0.48; 95% CI 0.30–0.77) [188].

For post-tuberculosis lung disease, international clinical standards call for assessment of rehabilitation needs based on persistent symptoms and functional limitations; these provisions are based on expert consensus [6]. In a single-center RCT by Akylbekov et al., conducted in Kyrgyzstan in 114 adults with blinded outcome assessment, a six-week program improved exercise tolerance and quality of life. The between-group difference in the increase in incremental shuttle walk distance was 123.0 m (95% CI 81.1–164.8). Participants were enrolled on the basis of post-tuberculosis lung disease after treatment completion, rather than confirmed COPD; muscle mass was not measured [74].

Findings in post-tuberculosis lung disease do not establish the effectiveness or safety of similar programs during active TB, nor do they determine when exercise should begin or its intensity in this population [6,74]. When organizing in-person sessions during treatment, the risk of infection transmission must be considered separately, and WHO infection prevention and control requirements must be followed, including administrative measures, ventilation, and respiratory protection [199].

### 7.4. From Nutritional Rehabilitation to Individualized Chronic Obstructive Pulmonary Disease Management

COPD is heterogeneous in its causes, clinical expression, and response to treatment [3,7], and its management is increasingly organized around treatable traits, that is, pulmonary, extrapulmonary, and behavioral characteristics that can be identified and targeted in individual patients [200,201]. In TB–COPD, malnutrition, low muscle mass and strength, deconditioning, and exertional hypoxemia can be regarded as extrapulmonary traits that are addressed together with pulmonary traits, such as airflow limitation, exacerbation risk, bronchiectasis, and blood eosinophil count, and with behavioral traits, such as smoking and physical inactivity. Treatment goals are also shifting from short-term symptom control toward sustained disease stability, with prevention of exacerbations and preservation of lung function [202]. For patients with muscle wasting, these goals are linked: exacerbations and hospitalizations are periods of accelerated muscle loss with incomplete recovery [66], and repeated courses of systemic glucocorticoids may add to muscle catabolism [76,182]. Because maintenance pharmacotherapy reduces the risk of exacerbations [3], it may also indirectly help to preserve muscle mass. Nutritional assessment and support start at TB diagnosis and continue throughout treatment [70]; pulmonary rehabilitation is offered to clinically stable patients and early after hospitalization for an exacerbation [188]; and in post-tuberculosis lung disease, rehabilitation needs are assessed after treatment completion [6,74]. Individual goals, for example, for body weight, handgrip strength, exercise capacity, and exacerbation frequency, can be recorded at baseline and reassessed at the end of TB treatment and during follow-up with the same methods (Section 6).

### 7.5. Treatment of Respiratory Disease and Treatment Safety

The TB treatment regimen is selected according to the organism’s drug susceptibility and the clinical characteristics of the disease [203]. Maintenance treatment for COPD is tailored to symptom burden and exacerbation history. Smoking cessation support, inhaler technique assessment, and evaluation of treatment adherence remain essential components of care [3].

Inhaled corticosteroids (ICS) are added to long-acting bronchodilators to prevent exacerbations, taking exacerbation frequency and blood eosinophil counts into account. In patients with concomitant asthma, ICS must be included in the treatment regimen [3]. A history of mycobacterial infection is considered when individually assessing the balance between expected benefit and infection risk [3]. In a South Korean cohort, after propensity score matching with 16,514 patients in each group, fluticasone propionate use was associated with a higher risk of TB than budesonide use (adjusted HR 1.28; 95% CI 1.05–1.60) [204]. Patients with previously recorded TB were excluded, so recurrence risk was not assessed. The observational design and lack of data on smoking and lung function limit causal interpretation of this association. These findings alone do not justify withdrawing ICS when indications for their use remain [3,204].

For COPD with severe chronic resting hypoxemia, the ATS recommends long-term oxygen therapy for at least 15 h per day (strong recommendation; moderate-certainty evidence) [205]. Long-term oxygen therapy is not routinely prescribed for moderate resting hypoxemia, whereas ambulatory oxygen is considered for severe exertional hypoxemia. Both recommendations are conditional and based on low-certainty evidence [205]. Indications for oxygen therapy are determined by the severity of hypoxemia; the cited evidence does not establish an independent effect on the recovery of muscle mass in patients with coexisting TB and COPD [205].

### 7.6. Limitations of the Evidence and This Review

None of the studies reviewed establishes the effectiveness of molecularly targeted interventions for muscle wasting in patients with coexisting TB and COPD. Clinical examples come from other patient populations. In a phase II trial involving 67 patients with COPD and low muscle mass, bimagrumab, which blocks activin type II receptors, increased thigh muscle volume, but no improvement in six-minute walk distance was detected over 24 weeks [147]. The primary endpoint was the change in thigh muscle volume measured by magnetic resonance imaging (MRI) at eight weeks; the trial was powered for this endpoint [147]. Thus, increased muscle volume in this trial was not accompanied by a demonstrated improvement in exercise tolerance. In a separate 12-week phase II trial involving 187 patients with cancer cachexia and GDF-15 concentrations of at least 1500 pg/mL, ponsegromab, an antibody against GDF-15, increased body weight compared with placebo [100]. These findings do not establish the drug’s effectiveness or safety in TB–COPD, and the GDF-15 threshold used in cancer cachexia cannot be considered a validated selection criterion for this population.

In the studies reviewed, modulation of sphingolipid metabolism to address muscle wasting was investigated only in cell and animal models [163,168]. In TB, this approach requires a separate safety assessment: sphingolipids participate in interactions between *M. tuberculosis* and macrophages, and the available evidence does not allow the direction of its effect on the course of infection to be predicted [176,177]. Anticytokine interventions targeting inflammation-driven catabolism also require a separate assessment of infection-related safety. TNF blockade increases the risk of active TB, primarily through reactivation of latent infection [86]. In studies of such interventions in TB–COPD, primary endpoints should include measures of muscle function, while effects on the course of infection should be assessed as a separate safety endpoint.

Direct studies in patients with TB–COPD, mostly with COPD after previous TB, are observational, and none of them reported measurements of muscle mass or strength [5,15,33,34]. None of the studies reviewed was a randomized trial of nutrition or rehabilitation in this population. The cited RCTs were conducted in TB without a separate analysis of coexisting COPD [71], in COPD without a separate analysis of TB history [72,73], and in post-tuberculosis lung disease without requiring confirmation of COPD [74]. WHO and ATS recommendations are also based predominantly on studies of these conditions separately [70,188]. For TB–COPD, these findings constitute indirect evidence; the magnitude of the effect in this population has not been established.

This gap could be addressed by prospective cohort studies of patients with COPD and newly diagnosed TB and of patients with COPD after previous TB, with TB-only and COPD-only comparison groups and with GLIM and EWGSOP2 or AWGS assessments at diagnosis, at treatment completion, and at 12 months. Another direction is RCTs of nutritional support and rehabilitation initiated during the continuation phase of TB treatment, with handgrip strength and appendicular lean mass as primary endpoints. Secondary endpoints could include COPD exacerbations, hospitalizations, treatment completion, and mortality. Analyses of prespecified subgroups with a history of TB in COPD nutrition and rehabilitation trials would also be useful. Such studies could include measurements of IL-6, GDF-15, myostatin, kynurenine, and ceramides to test their prognostic value in the clinical context. Infection prevention and control measures must be followed during in-person contact throughout treatment.

This is a narrative review. A systematic search and study selection protocol were not used, the quality of included studies was not formally assessed, and definitions of cachexia, sarcopenia, and malnutrition differed across the primary studies. Some mechanistic evidence comes from cell and animal models or from patients with COPD or malignancy. Selective citation is therefore possible, and quantitative comparisons across studies are not feasible.

## 8. Conclusions

Cachexia and sarcopenia in TB and COPD are clinically important conditions that require assessment irrespective of baseline BMI and the presence of disorders of glucose metabolism. Conventional metabolic assessment, focused on hyperglycemia and obesity, does not capture the full spectrum of abnormalities, because normal BMI and normal glycemia do not exclude a muscle deficit. Considering adiposity and catabolism as separate dimensions (Figure 1) helps to identify patients in whom obesity or diabetes coexists with muscle wasting.

Muscle wasting in TB and COPD has been linked to systemic inflammation, impaired energy metabolism and proteostasis, mitochondrial dysfunction, endocrine changes, and insufficient physical activity. The involvement of ceramides in these processes has been demonstrated predominantly in experimental models. The combined effects of these mechanisms in patients with both diseases remain insufficiently studied. In TB–COPD, more pronounced systemic inflammation and frequent hospitalizations, observed mainly in COPD after previous TB, and structural post-tuberculosis damage, observed in TB survivors, are the most plausible points of convergence, but none of these has yet been linked to measured muscle outcomes. The evidence base for patients with coexisting TB and COPD is limited, and most studies have examined each disease separately. The effectiveness of pharmacological agents targeting the molecular mechanisms of muscle wasting has not been established in this population.

Clinical management of patients with coexisting TB and COPD should include distinguishing post-tuberculosis airflow obstruction from COPD, diagnosis of malnutrition using the GLIM criteria, assessment of sarcopenia with population-appropriate criteria (EWGSOP2 or AWGS), assessment of respiratory limitations, and identification of reversible causes of deterioration. Nutritional support and pulmonary rehabilitation are provided alongside individualized treatment of the underlying disease. Treatment effectiveness is assessed through changes in muscle strength, physical performance, and body composition.

Priorities for future research include prospective assessment of muscle function in TB–COPD cohorts with TB-only and COPD-only comparison groups, development of personalized nutritional and rehabilitation strategies, and validation of biomarkers (IL-6, GDF-15, myostatin, kynurenine, and ceramides) for predicting muscle deficits and treatment response.

## Figures and Tables

**Table 1:** Selected operational definitions of sarcopenia and sarcopenic obesity.

Definition	Population	Diagnostic Components	Selected Cutoffs
EWGSOP2 [26]	Older adults; European reference data	Low muscle strength: probable sarcopenia; plus low muscle quantity or quality: confirmed sarcopenia; plus low physical performance: severe sarcopenia	Handgrip strength <27 kg (men) and <16 kg (women); five chair rises >15 s; appendicular muscle mass index (DXA) <7.0 and <5.5 kg/m^2^; gait speed ≤0.8 m/s or SPPB score ≤8 (severe sarcopenia)
AWGS 2019 [29]	Older adults; Asia	Low muscle mass plus low muscle strength or low physical performance; “possible sarcopenia” (low strength or low performance) for primary care	Handgrip strength <28 kg (men) and <18 kg (women); five chair stands ≥12 s; 6 m gait speed <1.0 m/s; SPPB score ≤9; appendicular muscle mass index <7.0 and <5.4 kg/m^2^ (DXA) or <7.0 and <5.7 kg/m^2^ (bioimpedance)
AWGS 2025 [30]	Adults aged ≥50 years; Asia	Low muscle mass plus low muscle strength; physical performance treated as an outcome; BMI-adjusted muscle mass index added	Adults aged 50–64 years: handgrip strength <34 kg (men) and <20 kg (women); height-adjusted appendicular muscle mass index <7.2 (men) and <5.5 kg/m^2^ (women) by DXA or <7.6 and <5.7 kg/m^2^ by bioimpedance. Adults aged ≥65 years: AWGS 2019 cutoffs for handgrip strength and height-adjusted muscle mass retained. No thresholds for adults younger than 50 years; not to be applied automatically to younger patients (Section 6.2)
SDOC [31]	Older adults	Weakness (low grip strength) and slowness (low usual gait speed); lean mass measured by DXA not included	See the position statements
GLIS [32]	Global conceptual definition	Components: muscle mass, muscle strength, and muscle-specific strength; impaired physical performance treated as an outcome	Operational cutoffs not yet defined
ESPEN/EASO sarcopenic obesity [35]	Adults	Screening: high BMI or waist circumference; diagnosis: impaired muscle function plus altered body composition (high fat mass and low muscle mass relative to body weight)	See the consensus statement

Abbreviations: AWGS, Asian Working Group for Sarcopenia; BMI, body mass index; DXA, dual-energy X-ray absorptiometry; EASO, European Association for the Study of Obesity; ESPEN, European Society for Clinical Nutrition and Metabolism; EWGSOP2, European Working Group on Sarcopenia in Older People 2; GLIS, Global Leadership Initiative in Sarcopenia; SDOC, Sarcopenia Definition and Outcomes Consortium; SPPB, Short Physical Performance Battery.

**Table 3:** Principal mechanisms of muscle wasting and the nature of the supporting evidence, including data in TB–COPD. The last column specifies the population studied: direct data come mainly from patients with COPD after previous TB, and no study has analyzed active TB with COPD separately.

Process	Potential Consequence	Nature of the Evidence	Data in TB–COPD
Systemic inflammation	Reduced appetite and increased catabolism	Clinical studies in TB; muscle biopsies in COPD; experimental models [50,51,69,80,88]	COPD after TB: higher CRP, erythrocyte sedimentation rate, and IL-6 than in smoking-related COPD [68]. Previous or current TB with airflow obstruction: systemic inflammatory response index distinguished patients with and without obstruction [79]. Muscle not assessed in either study
Impaired proteostasis	Reduced muscle protein synthesis and increased breakdown	Protein metabolism in active TB; muscle proteolysis and autophagy pathways in COPD [51,105,106,144]	Not studied
Mitochondrial dysfunction	Impaired energy supply and oxidative damage	Muscle biopsies in COPD; predominantly infected macrophage models in TB [89,127,128,129,130,131]	Not studied
Altered adipose tissue and endocrine regulation	Impaired appetite and body composition recovery	Clinical observations in TB and COPD; causal relationships have not been established for all measures [18,19,50,77,156]	COPD with previous or active TB (analyzed together): obesity and diabetes recorded less often than in other patients with COPD [15]
Altered ceramide metabolism	Altered insulin response, proteostasis, and mitochondrial respiration	Predominantly cellular and animal models; studies in patients with COPD have examined lung tissue and blood plasma [163,168,172,173,174,175,176,177]	Not studied
Low physical activity and recurrent exacerbations	Deconditioning and incomplete muscle recovery	Longitudinal observations and muscle tissue studies in COPD [66,106,131,180]	COPD after TB: more frequent hospitalizations and, in some studies, exacerbations than in other patients with COPD [5,15,33,34]
Tryptophan–kynurenine pathway	Muscle weakness and frailty (hypothesis)	Kynurenine associated with weaker grip strength and frailty in older adults; not a TB or COPD population [81]	COPD after TB: higher kynurenine and lower serotonin than in smoking-related COPD [78]
Structural post-tuberculosis damage	Exertional hypoxemia, recurrent infection, and exercise limitation	Indirect: low muscle mass (termed sarcopenia by the authors) more likely with residual radiographic changes in TB survivors [56]; lower muscle strength and function in post-tuberculosis lung disease [59]	General COPD cohort: tuberculosis-destroyed lung more frequent in patients with low BMI [24]

Abbreviations: BMI, body mass index; COPD, chronic obstructive pulmonary disease; CRP, C-reactive protein; IL-6, interleukin 6; TB, tuberculosis; TB–COPD, coexisting tuberculosis and chronic obstructive pulmonary disease.

## Data Availability

No new data were created or analyzed in this study. Data sharing is not applicable to this article.

## Abbreviations

The following abbreviations are used in this manuscript:

Akt	protein kinase B
AMPK	adenosine monophosphate-activated protein kinase
ATP	Adenosine triphosphate
ATS	American Thoracic Society
AWGS	Asian Working Group for Sarcopenia
BMI	Body mass index
CHS	Cardiovascular Health Study
CI	Confidence interval
COPD	Chronic obstructive pulmonary disease
CRP	C-reactive protein
CT	Computed tomography
DKK3	Dickkopf-related protein 3
DNA	deoxyribonucleic acid
DXA	Dual-energy X-ray absorptiometry
EASO	European Association for the Study of Obesity
ESPEN	European Society for Clinical Nutrition and Metabolism
EWGSOP	European Working Group on Sarcopenia in Older People, original consensus
EWGSOP2	European Working Group on Sarcopenia in Older People, revised consensus
FEV_1_	Forced expiratory volume in one second
FFMI	fat-free mass index
FGF-21	fibroblast growth factor 21
FOXO	forkhead box O
FOXO1	forkhead box O1
FOXO3	forkhead box O3
FVC	Forced vital capacity
GDF-15	Growth differentiation factor 15
GLIM	Global Leadership Initiative on Malnutrition; international criteria for malnutrition
GLIS	Global Leadership Initiative in Sarcopenia
GOLD	Global Initiative for Chronic Obstructive Lung Disease
HbA1c	Glycated hemoglobin
HIF-1	hypoxia-inducible factor 1
HIF-1α	hypoxia-inducible factor 1 alpha
HIV	Human immunodeficiency virus
HMGB1	high-mobility group box 1
HR; aHR	Hazard ratio; adjusted hazard ratio
ICS	Inhaled corticosteroids
IFN-γ	interferon gamma
IGF-1	insulin-like growth factor 1
IKK-α	inhibitor of nuclear factor kappa B kinase subunit alpha
IKKβ	inhibitor of nuclear factor kappa B kinase subunit beta
IL-6	Interleukin 6
JAK	Janus kinase
LC3B	microtubule-associated protein 1 light chain 3 beta
LC3B-II	the lipidated form of LC3B
LDHA	lactate dehydrogenase A
MOTS-c	mitochondrial open reading frame of the 12S ribosomal ribonucleic acid-c
mRNA	messenger ribonucleic acid
mtDNA	Mitochondrial DNA
mTOR	mechanistic target of rapamycin
mTORC1	mechanistic target of rapamycin complex 1
MuRF1	muscle RING finger protein 1
NAD^+^	oxidized nicotinamide adenine dinucleotide
NF-κB	nuclear factor kappa-light-chain-enhancer of activated B cells
NRF1	nuclear respiratory factor 1
OR; aOR	Odds ratio; adjusted odds ratio
OXPHOS	Oxidative phosphorylation
p70S6K	70-kDa ribosomal protein S6 kinase
PGC-1α	peroxisome proliferator-activated receptor gamma coactivator 1 alpha
PI3K	phosphoinositide 3-kinase
PTLD	Post-tuberculosis lung disease
RCT	Randomized controlled trial
REDD1	regulated in development and DNA damage responses 1
ROS	Reactive oxygen species
RR	Relative risk
SARC-F	A questionnaire for identifying sarcopenia risk
SDOC	Sarcopenia Definition and Outcomes Consortium
SMAD2/3	SMAD family members 2 and 3
SPPB	Short Physical Performance Battery
STAT3	signal transducer and activator of transcription 3
TB	Tuberculosis
TB–COPD	Coexisting tuberculosis and chronic obstructive pulmonary disease
TFAM	mitochondrial transcription factor A
TGF-β	transforming growth factor beta
TNF	tumor necrosis factor
TNF-α	tumor necrosis factor alpha
WHO	World Health Organization
